# GHCU, a Molecular Chaperone, Regulates Leaf Curling by Modulating the Distribution of KNGH1 in Cotton

**DOI:** 10.1002/advs.202402816

**Published:** 2024-04-26

**Authors:** Yihao Zang, Chenyu Xu, Lishan Yu, Longen Ma, Lisha Xuan, Sunyi Yan, Yayao Zhang, Yiwen Cao, Xiaoran Li, Zhanfeng Si, Jieqiong Deng, Tianzhen Zhang, Yan Hu

**Affiliations:** ^1^ Zhejiang Provincial Key Laboratory of Crop Genetic Resources Institute of Crop Science Plant Precision Breeding Academy College of Agriculture and Biotechnology Zhejiang University Zhejiang 310058 China; ^2^ Industrial Crop Research Institute Sichuan Academy of Agricultural Sciences Sichuan 610066 China; ^3^ Hainan Institute of Zhejiang University Sanya 572025 China

**Keywords:** auxin, cup‐shaped leaf, *Gossypium hirsutum*, map‐based cloning

## Abstract

Leaf shape is considered to be one of the most significant agronomic traits in crop breeding. However, the molecular basis underlying leaf morphogenesis in cotton is still largely unknown. In this study, through genetic mapping and molecular investigation using a natural cotton mutant *cu* with leaves curling upward, the causal gene *GHCU* is successfully identified as the key regulator of leaf flattening. Knockout of *GHCU* or its homolog in cotton and tobacco using CRISPR results in abnormal leaf shape. It is further discovered that GHCU facilitates the transport of the HD protein KNOTTED1‐like (KNGH1) from the adaxial to the abaxial domain. Loss of GHCU function restricts KNGH1 to the adaxial epidermal region, leading to lower auxin response levels in the adaxial boundary compared to the abaxial. This spatial asymmetry in auxin distribution produces the upward‐curled leaf phenotype of the *cu* mutant. By analysis of single‐cell RNA sequencing and spatiotemporal transcriptomic data, auxin biosynthesis genes are confirmed to be expressed asymmetrically in the adaxial‐abaxial epidermal cells. Overall, these findings suggest that GHCU plays a crucial role in the regulation of leaf flattening through facilitating cell‐to‐cell trafficking of KNGH1 and hence influencing the auxin response level.

## Graphical abstract

Leaf flattening depends on the establishment of adaxial‐abaxial polarity, which process is controlled by a complex regulatory network. Here, it is reported that the GHCU can transport KNGH1 to the abaxial boundary cells; as KNGH1 inhibits the auxin response, this leads to higher auxin content on the abaxial side compared to the adaxial side.

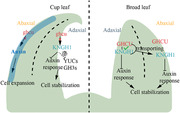

## 1 Introduction

Plants have evolved various leaf shapes in the course of adapting to different ecological environments. Some plants have deeper lobes to protect themselves from insects or intense sunlight, while others have larger surface areas to maximize sunlight absorption. During development, a leaf blade undergoes a series of processes that include founder cell recruitment, distal growth, blade initiation, and intercalary growth.^[^ ^1^ ^]^ The shoot apical meristem (SAM) continuously produces young leaves that emerge from the flank of the meristem as dorsiventral structures. Subsequently, leaf primordia develop patterns to form planar leaves, including dorsoventral (adaxial‐abaxial in plants), proximodistal, and mediolateral patterns. Throughout the process of leaf morphogenesis, the regulation of transcription factors, small RNAs, and hormones is tightly coordinated.^[^ ^2^ ^]^

Leaf flattening depends on the establishment of adaxial‐abaxial polarity, which process is controlled by a complex regulatory network.^[^ ^3^ ^]^ Any defect or disruption in the establishment of adaxial‐abaxial polarity will result in the rolling or curly‐leaf shape in plants. Proper leaf curling contributes to a more erect plant structure, leading to enhanced canopy light capture and reduced water loss via transpiration, which in turn improves photosynthetic efficiency and resistance to environmental stress.^[^ ^4^, ^5^, ^6^ ^]^ Consequently, modest leaf blade curling is considered an important agronomic trait in breeding for high yield.^[^ ^5^, ^7^, ^8^ ^]^ Although several genetic regulators of leaf curling have been characterized in rice and maize, such as *RLD1*, *SFL1*, and *OsZHD1*,^[^ ^9^, ^10^, ^11^ ^]^ no genes related to rolling leaf phenotypes have been reported in cotton. Therefore, investigation of the genes that control leaf curling may be beneficial in breeding crops with desired architecture.^[^ ^12^ ^]^

Many plant developmental processes, including leaf formation, require mobile transcriptional regulators to transmit essential positional information.^[^ ^13^, ^14^, ^15^, ^16^ ^]^ For instance, the maize homeodomain (HD) protein KNOTTED1 (KN1) was the first plant protein reported to traffic between cells.^[^ ^17^ ^]^ *KN1* is primarily expressed in layer 2 SAM cells, but its protein can be detected in non‐expressing cells, suggesting its ability to move within the SAM.^[^ ^18^ ^]^ In *Arabidopsis*, studies have shown that the KN1 orthologues STM and KNAT1 give cell‐autonomous proteins a gain‐of‐trafficking function in SAM cells, leaves, and the stem.^[^ ^19^, ^20^ ^]^ HD proteins belonging to the KNOTTED‐like homeobox domain (KNOX) family in *Arabidopsis*, *Zea mays*, and *Physcomitrella* also exhibit intercellular trafficking activity.^[^ ^19^, ^21^, ^22^ ^]^ Ultimately, members of this family share an HD that is crucial for cell‐to‐cell movements and performs intercellular trafficking through the plasmodesmata (PD), suggesting that plants have developed a unique mechanism for transporting KNOX proteins. However, it remains unknown how HD proteins move from cell to cell.

A natural curly leaf mutant called “*cu*”, discovered by Mr. H.C. Kuo in 1928, exhibits an upward‐curling margin that resembles a cup.^[^ ^23^ ^]^ Despite the age of this mutant, the gene responsible for this trait has not yet been identified; the molecular basis for this cup‐shaped leaf is also yet unclear. Here, we investigated the *cu* mutant to gain deeper insights into the transcription regulation network involved in leaf development. We employed the map‐based cloning method to identify the causal gene underlying the *cu* locus, which encodes a chaperonin containing TCP‐1 8 (CCT8) subunit and is named *GHCU*. A series of transgenic plant experiments confirmed GHCU to be the essential regulator responsible for the formation of cup‐shaped leaves in the *cu* mutant. We also proposed a possible mechanism for *GHCU* involvement in leaf development that is congruent with our experimental data.

## 2 Results

### 2.1 Map‐Based Cloning of the *cu* Locus

**Figure 1:**
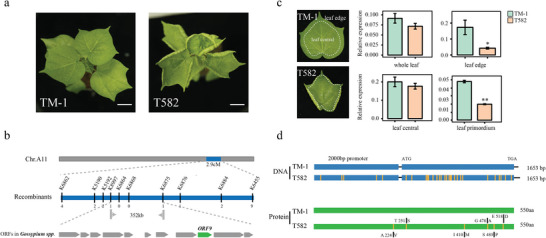
Cloning of the cu locus. a) Phenotypes of TM‐1 and T582. Scale bars = 5 cm. b) Using the F_2_ and BC_1_ generations, *cu* was fine‐mapped to a 352‐kb interval on chromosome A11 between k8997 and k6875. Within that region, ORF9 (*GH_A11G3380*) was selected as a candidate gene. c) Relative expression of *GH_A11G3380* in whole leaf, leaf margin, leaf center, and leaf primor‐dium of TM‐1 and T582 as determined by qRT‐PCR (^*^ *p* < 0.05, ^**^ *p* < 0.01, *t*‐test). The white dotted line indicates the sampling boundary. d) Schematic representation of GH_A11G3380 DNA and protein sequences. Yellow lines on T582 DNA indicate SNP positions in the promoter and the ORF; Yellow lines on the T582 protein indicate amino acid mutations.

In cotton plants, leaf morphology plays a critical role in determining fiber yield and quality. Texas 582 (T582) is a multiple‐recessive marker line that was developed by simultaneously introducing five recessive mutant genes into TM‐1, a standard genetic line of upland cotton,^[^ ^24^, ^25^ ^]^ including the cup leaf locus called *cu*. Compared with TM‐1, T582 exhibits leaves that are adaxially rolled at the leaf margin; however, the overall leaf area at a given age is similar to TM‐1 (**Figure** **1**a). Observation using scanning electron microscopy (SEM) revealed that T582 has an abnormal arrangement of enlarged epidermal cells on the abaxial side compared with TM‐1, though they have similar leaf areas (Figure S1a,b, Supporting Information). Similar results were also observed in paraffin sections (Figure S1c, Supporting Information). Furthermore, SEM analysis of young leaves showed that the leaf shape is already determined at the incipient leaf stage (Figure S1d, Supporting Information).

Our previous study primarily anchored the *cu* locus on a 2‐Mb interval of chromosome A11 through bulked‐segregant analysis and sequencing (BSA‐seq).^[^ ^25^ ^]^ To further isolate the *cu* gene, we performed a cross between T582 and TM‐1 to construct an F_2_ population and a BC_1_ population for genetic analysis and gene mapping. The populations exhibited phenotypic segregation indicating that *cu* is inherited as a single recessive mutation (Table S1, Supporting Information) (F_2_: 1708 flat‐ and 492 cup‐leaved plants, fitting the expected 3:1 ratio: χ^2^ = 0.0152 < χ^2^_0.05_ = 3.84, df = 1; BC_1_: 207 flat‐ and 212 cup‐leaved plants, fitting the expected 1:1 ratio: χ^2^ = 0.0382 < χ^2^_0.05_ = 3.84, df = 1).^[^ ^26^ ^]^ Of the 3324 molecular markers developed in our laboratory,^[^ ^27^ ^]^ over 200 polymorphic markers were screened out between TM‐1 and T582. These polymorphic markers were applied to screen 492 F_2_ individuals with cup‐shaped leaves, which successfully narrowed down the *cu* locus to within a 352‐kb region flanked by the markers K8997 and K6875 (Figure 1b). Based on the gene annotation of the TM‐1 genome,^[^ ^28^ ^]^ 11 putative candidate genes are predicted in this interval (Table S2, Supporting Information). The expression patterns of these genes were examined based on transcriptomic data from nine tissues of TM‐1 ^[^ ^28^ ^]^ (Table S3, Supporting Information). Among the 11 candidate genes, seven were found to be expressed in leaves (defined as transcripts per million [TPM] >1), and their expression in the leaf edge and leaf primordium was confirmed by real‐time quantitative polymerase chain reaction (qRT‐PCR) (Figure S2, Supporting Information). A comparison of expression levels between TM‐1 and T582 revealed only one gene with differential expression in the leaf edge, while four genes showed differential expression in the leaf primordium. Notably, of special interest was *ORF9* (*GH_A11G3380*), which was differentially expressed in both tissues (Figure 1c; Figure S2, Supporting Information).

To further evaluate gene function in leaf development, the seven leaf‐expressed ORFs (ORF1, 2, 5, 6, 7, 8, and 9) were knocked down by virus‐induced gene silencing (VIGS). Plants in which *GH_A11G3380* was silenced exhibited newly growing leaves with a crinkled leaf blade toward the abaxial side (Figure S3a–c, Supporting Information). Paraffin sections and SEM further showed cell enlargement on the abaxial epidermis, while the abaxial epidermal cells were irregularly arranged compared with the control group (Figure S3d,e, Supporting Information). Notably, cell enlargement on the abaxial side of the silenced group was identical to the cup leaf mutant T582, while having no significant difference in leaf area (Figure S3f, Supporting Information). All these results strongly support that *GH_A11G3380* is the causal gene underlying the *cu* locus. *GH_A11G3380* is annotated as a chaperonin containing TCP‐1 subunit and is hereafter renamed *Gossypium hirsutum cup‐shaped leaf gene* (*GHCU*). The full‐length coding region and the 2.0 kb region upstream of *GHCU* were isolated and sequenced from both TM‐1 and T582. Sequence alignment detected 24 single nucleotide polymorphisms (SNPs) conferring six altered amino acids in T582 compared to TM‐1 (Figure 1d).

*GHCU* is constantly expressed in all examined vegetative and reproductive organs, as demonstrated by qRT‐PCR. Slightly higher expression levels were detected in roots and fibers, but no significant difference between TM‐1 and T582 was found in those tissues (Figure S4a, Supporting Information). To gain a more detailed understanding of *GHCU* expression, we fused a ≈2.0‐kb fragment of the *GHCU* promoter with the *Escherichia coli* β‐glucuronidase (GUS) reporter gene and introduced it into *Arabidopsis* to generate the stable transgenic plants. GUS activity was observed in leaves, stems, roots, and inflorescence stems (Figure S4b–e, Supporting Information), supporting its widespread expression. RNA in situ hybridization analysis provided a detailed perspective of relatively stronger expression of *GHCU* in the SAM and young leaves (Figure S4f, Supporting Information).

### 2.2 Loss‐of‐Function Mutation of *GHCU* Leads to the Cup‐Shaped Leaf Phenotype in Transgenic Plants

**Figure 2:**
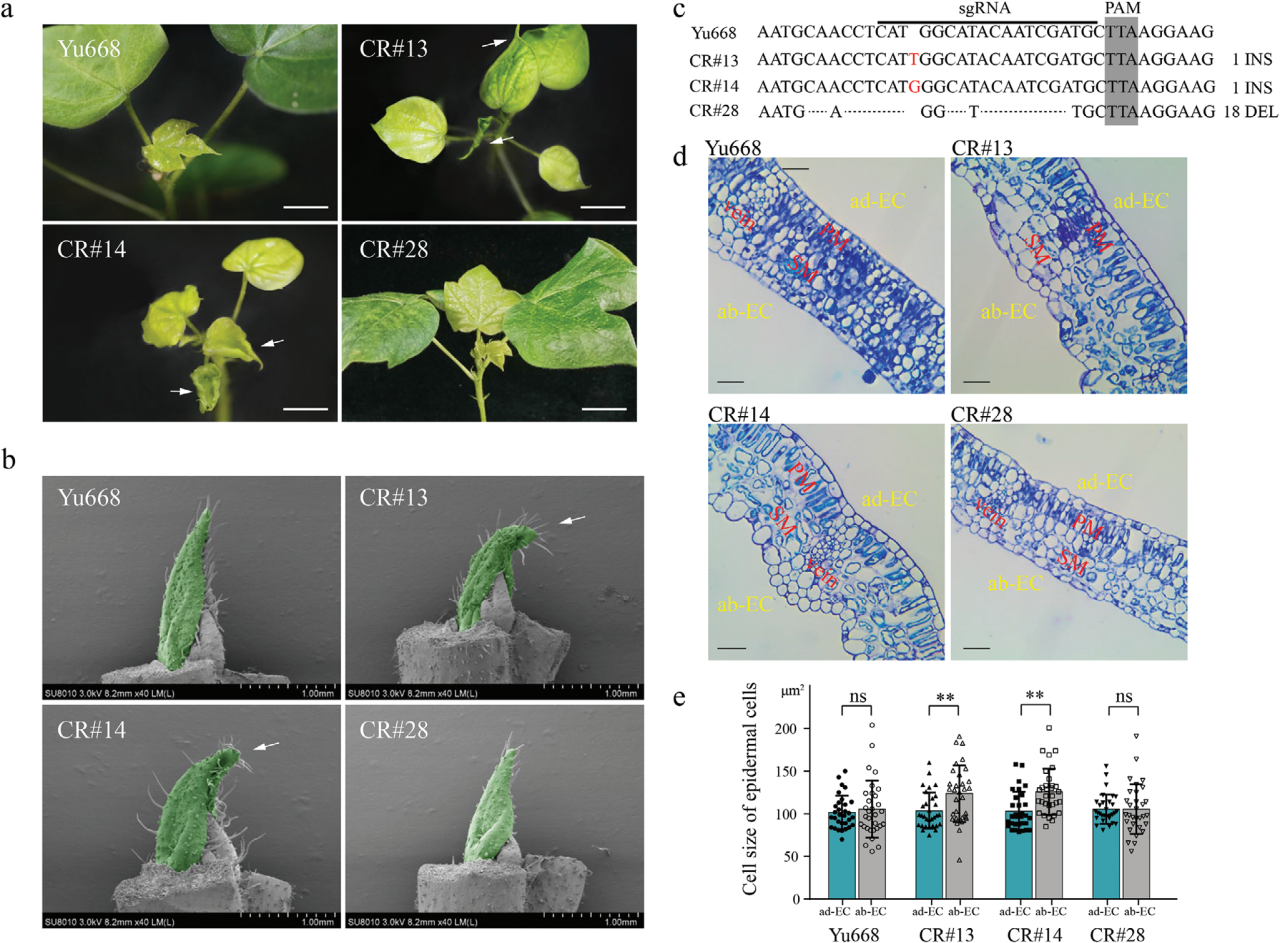
Phenotypic analysis of *GHCU* knockdown transgenic plants *Gossypium hirsutum*. a) Images of young leaves of wild‐type (Yu668), *GHCU* gene‐editing plants CR#13, CR#14, and CR#28. b) SEM images of the leaf primordium. The 1st true leaf of Yu668 and *GHCU* gene‐editing plants were painted green. Scale bars = 100 µm. c) Sequences around the target sites in the CRISPR/Cas9 cotton mutants. The dashed line indicates deletion and the red letter indicates insertion. The horizontal lines refer to sgRNA sequences. PAM sequences are shaded in gray. d) Toluidine blue staining of paraffin sections of Yu668, CR#13, CR#14, and CR#28, ad‐EC: adaxial epidermal cell, ab‐EC: abaxial epidermal cell, PM: Palisade mesophyll cells, SM: Spongy mesophyll cells. Scale bars = 20 µm. e) Cell size of epidermal cells in (d). *n* > 30, *p*‐values were determined by the Student's *t*‐test (^**^ *p* < 0.01, *t*‐test).

To test whether the mutation of *GHCU* is responsible for the cup‐shaped phenotype of the *cu* mutant, we generated *GHCU* gene‐edited plants. A CRISPR/Cas9 plasmid containing a *GHCU*‐specific guide RNA driven by the U6 promoter was constructed and transformed into the cotton cultivar Yu668 using *Agrobacterium tumefaciens*‐mediated transformation. A total of ten independent transgenic cotton lines were generated, the majority of which exhibited a cup‐shaped leaf phenotype similar to T582. Three homozygous transgenic lines, CR#13, CR#14, and CR#28, were selected for further analysis (**Figure** **2**a,b). Hi‐Tom sequencing revealed that CR#13 and CR#14 carried a 1‐bp insertion in the open reading frame region of *GHCU*, resulting in a frame shift. These lines showed leaf abnormalities with strong curling at the edges, which was totally different from the broad and flattened leaves of Yu668 plants. The third selected transgenic line CR#28 had an 18‐bp deletion in the ORF region that led to a six‐amino‐acid deletion (Figure 2c). It displayed normal leaves like the receptor, presumably due to this deletion, being an integral multiple of three, inducing only slight changes in the GHCU protein. We further examined the abaxial and adaxial leaf epidermis of transgenic and receptor plants using paraffin sections. This revealed CR#13 and CR#14 plants to have significantly enlarged cells in the abaxial epidermis at the leaf margins compared with the receptor Yu668 (Figure 2d,e). In addition, we generated seven transgenic lines in which *GHCU* was overexpressed under the control of the CMV35S promoter. All seven of these lines displayed normal leaf blades like wild‐type plants (Figure S5, Supporting Information). Finally, we created transgenic *Arabidopsis* plants by introducing *GHCU* into the *cct8* mutant (*SALK_082168c*), which has cup‐shaped leaves. With ectopic expression of *GHCU*, the leaves regained their normal shape (Figure S6, Supporting Information). These results support that *GHCU* plays a vital role in the formation of a flattened leaf blade.

**Figure 3:**
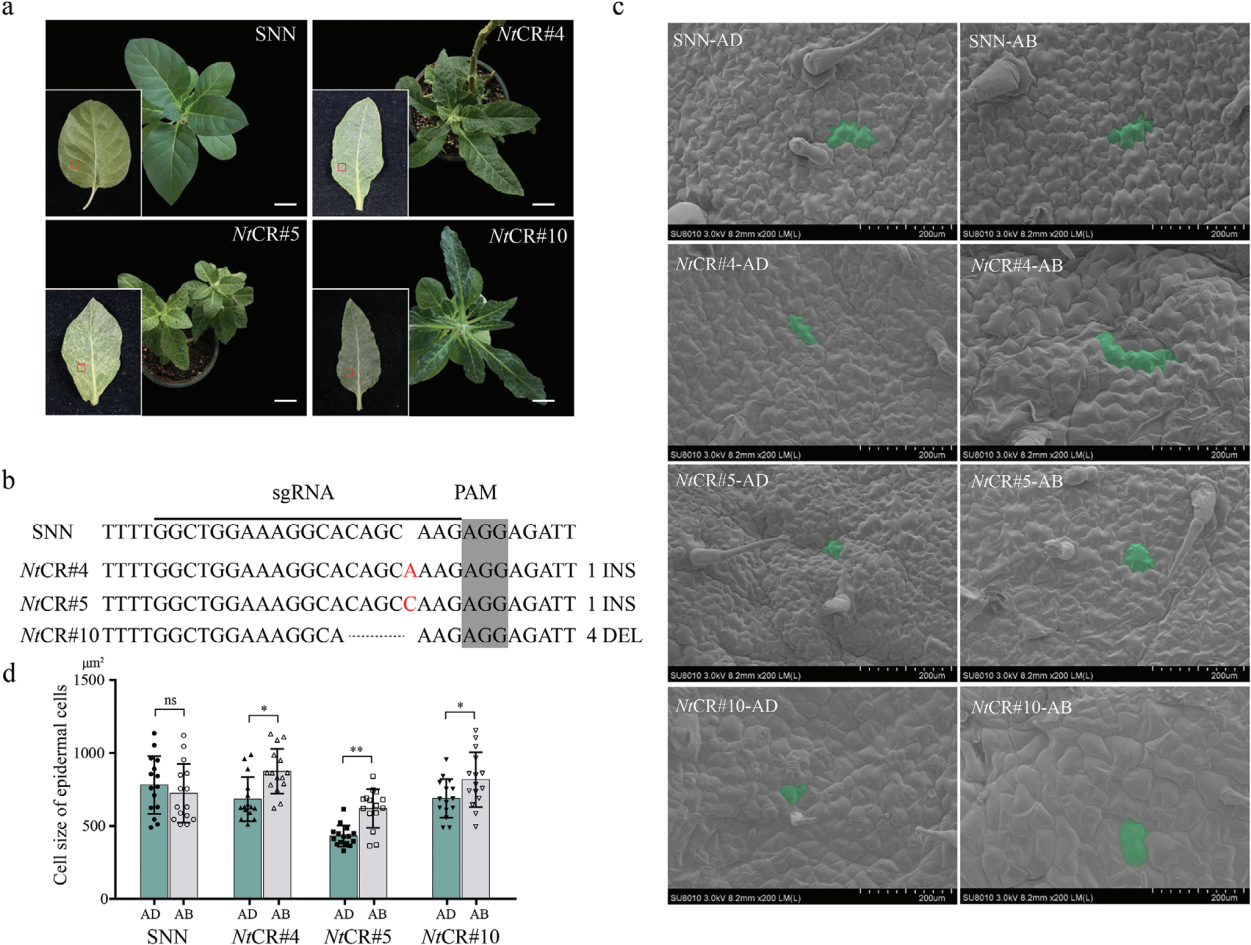
Phenotypic analysis of *GHCU*‐homologous gene *LOC107789350* knockdown transgenic tobacco plants. a) Images of wild‐type (SNN), tobacco gene‐editing plant *NtCR#4*, *NtCR#5*, and *NtCR#10*. b) Sequences around the target sites in the CRISPR/‐ Cas9 tobacco plants. The dashed line indicates deletion and the red letter indicates insertion. The horizontal lines refer to sgRNA sequences. PAM sequences are shaded in grey. c) SEM images of the surfaces of plants in the red box of (a). AD, adaxial side of the leaf. AB, abaxial side of the leaf. d) Cell size of epidermal cells in (c). *n* = 15. Scale bars = 3 cm in (a), 200 µm in (c). *p*‐values were determined by the Student's *t*‐test (^*^ *p* < 0.05; ^**^ *p* < 0.01, *t*‐test).

To test whether *GHCU* plays similar roles in other plants, we generated gene‐edited tobacco plants with knocked‐out *LOC107789350*, the homolog of *GHCU*. We found the majority of *LOC107789350*‐edited tobacco plants to exhibit abnormal leaf shapes compared to the control group (**Figure** **3**a). Three homozygous tobacco transgenic lines were selected for further analysis, each carrying a different mutation (1‐bp insertion, 1‐bp insertion, and 4‐bp deletion) in the CDS region of *LOC107789350* (Figure 3b). All of these gene‐edited plants displayed elongated leaves with a crinkled epidermis, which was distinct from the smooth and well‐organized epidermis of wild‐type tobacco. Scanning electron microscopy (SEM) revealed abnormal epidermal cells on the abaxial‐adaxial plane (Figure 3c); specifically, the abaxial side of the leaf had significantly larger epidermal cells compared to the adaxial side (Figure 3d). Together, these data suggest that *GHCU* and its homologs are key in regulating cell enlargement and arrangement on the abaxial epidermis during leaf development.

### 2.3 Loss of Function of GHCU and Its Homologs Reduces Leaf Indole‐3‐Acetic Acid (IAA) Content

Plant hormone distribution in tissues is known to be associated with cell proliferation. To investigate the role of plant hormones in leaf shape, we evaluated hormone content in leaves of transgenic and wild‐type tobacco using ESI‐HPLC‐MS/MS. Among the three plant hormones that were tested (indole‐3‐acetic acid [IAA], cytokinin, gibberellic acid_3/4_), only IAA showed significant variation (Figure S7a, Supporting Information), with *LOC107789350*‐edited plants exhibiting a significant reduction (Figure S7b, Supporting Information). Similarly, *GHCU*‐edited cotton lines CR#13 and CR#14 exhibited decreased IAA content, which correlated with the degree of leaf curliness (Figure S7c, Supporting Information). Overexpression of *GHCU* in tobacco resulted in normal leaf shape; however, the increase in *GHCU* transcripts did not affect IAA content (Figure S8, Supporting Information). These findings suggest that excess *GHCU* does not impact leaf development. However, loss of *GHCU* and its homologs such as *LOC107789350* in tobacco may lead to abnormal leaf development by reducing IAA content.

### 2.4 GHCU Physically Interacts with KNGH1

To investigate the transcriptional regulator network of *GHCU* in leaf development, a yeast two‐hybrid (Y2H) assay was performed using full‐length GHCU_TM‐1_ as the bait to identify its interacting proteins. Y2H assay showed GHCU to interact with KNOTTED1‐like homeobox protein, an ortholog of KNAT1 (Table S4, Supporting Information). The class I KNOTTED1‐like homeobox gene family consists of four members in *Arabidopsis*, of which SHOOT MERISTEMLESS (STM), BREVIPEDICELLUS (BP)/KNAT1, and KNAT2 have been proven to play important roles in the development of meristematic potential or leaf organogenesis.^[^ ^29^, ^30^, ^31^ ^]^ We constructed a phylogenic tree using the amino acid sequences of KNOTTED1‐like and other HD proteins in *Arabidopsis*; this revealed KNOTTED1‐like to cluster with the KNAT1 protein (Figure S9, Supporting Information). Accordingly, it was renamed as the protein KNOTTED1 in *Gossypium hirsutum* (KNGH1).

**Figure 4:**
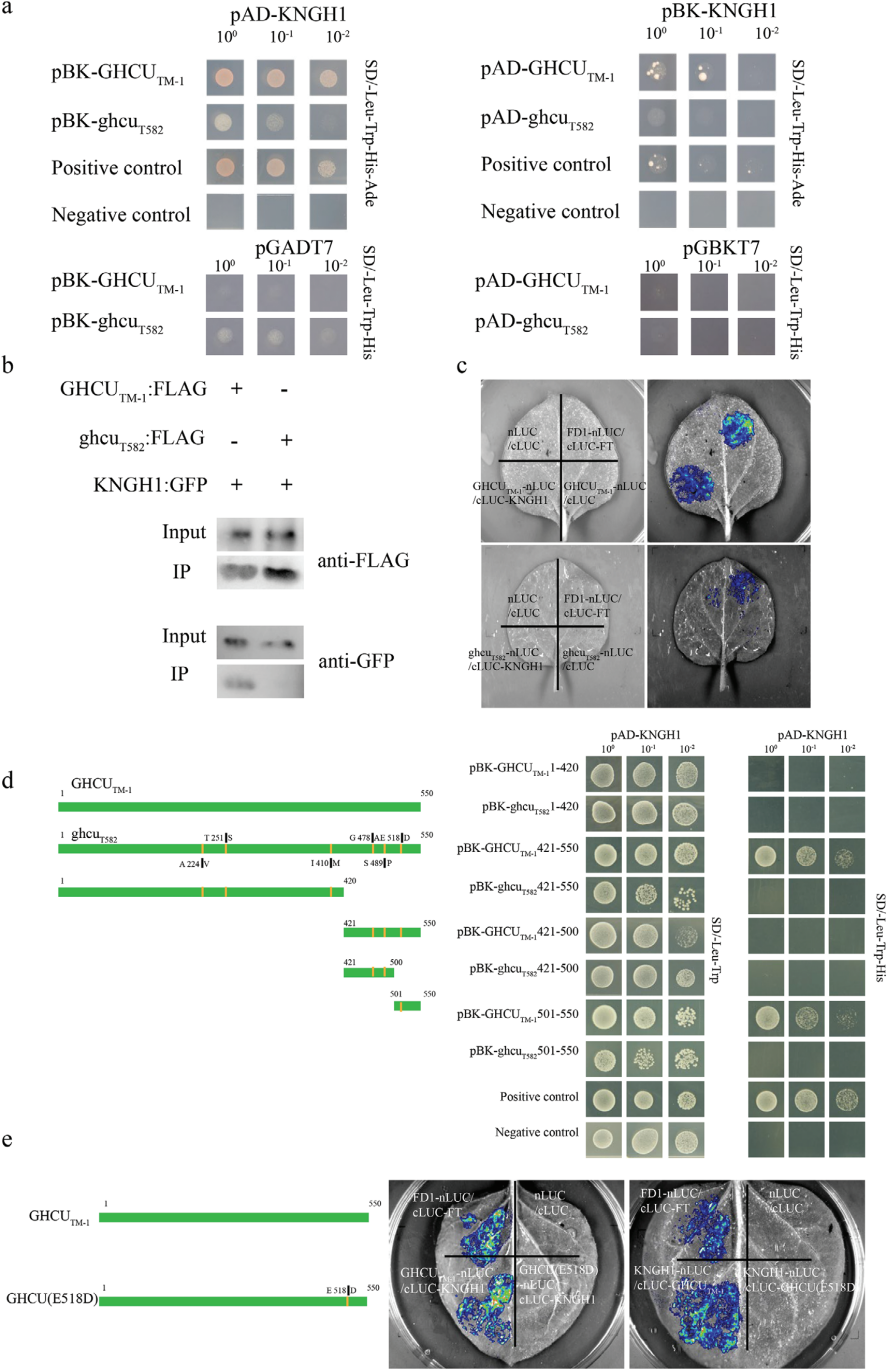
Interaction between GHCUTM‐1 and KNGH1. a) Y2H assays investigating the interaction between GHCU and KNGH1. GHCUTM‐1, but not ghcuT582, can interact with GHKN1, as indicated by (b) co‐IP and (c) split firefly luciferase complementation imaging assay. Co‐IP was carried out with anti‐FLAG agarose on total isolated proteins, and immunoblotting analysis was done with anti‐GFP antibodies. d) Y2H assays to investigate the interaction between different fragments of GHCU and KNGH1. C‐terminal amino acids of GHCU contribute to the interaction with KNGH1. e) GHCU, but not GHCU(E518D), can interact with KNGH1, as indicated by split firefly luciferase complementation imaging assay. pGBKT7‐p53 and pGADT7‐Tantigen were used as positive controls, and pGBKT7‐Lamin c and pGADT7‐Tantigen as negative controls.

Next, we used the Y2H assay to test the interaction of KNGH1 with GHCU from TM‐1 (GHCU_TM‐1_) and T582 (ghcu_T582_). It was observed that KNGH1 interacted strongly with GHCU_TM‐1_, but very weakly with the mutated form ghcu_T582_ (**Figure** **4**a). Both co‐immunoprecipitation (Co‐IP) and luciferase complementation imaging (LCI) assays likewise demonstrated direct interaction of KNGH1 with GHCU_TM‐1_ but not with ghcu_T582_ (Figure 4b,c). The interaction between ghcu_T582_ and KNGH1 might have been too weak to be detectable by Co‐IP and LCI.

To clarify the exact binding region through which GHCU and KNGH1 interact, GHCU was divided into several parts and employed in Y2H and LCI assays. Truncation analysis revealed the main recognition site for KNGH1 to be in the region of GHCU spanning 1,503 bp to 1,650 bp; this region contained the G_TM‐1_ to T_T582_ mutation, which causes an amino acid substitution from Glu^518^(E) to Asp^518^(D) (Figure 4d). Moreover, to verify whether this site mutation is critical for the interaction between GHCU and KNGH1, we mutated the Glu^518^ of GHCU_TM‐1_ to Asp^518^ [GHCU(E518D)] and performed an LCI assay. As expected, the mutated GHCU(E518D) did not interact with KNGH1 (Figure 4e), indicating this site has functional significance.

### 2.5 KNGH1 Regulates Auxin Response by Cell‐to‐Cell Tracking

From the above results, we concluded that the interplay of GHCU with KNGH1 regulates leaf flattening. To further explore the function of *KNGH1*, we induced its ectopic expression in *Nicotiana benthamiana*. Plants overexpressing *KNGH1* exhibited shrunken leaves in comparison with nontransgenic plants and significantly decreased IAA content as well (Figure S10, Supporting Information). This indicates that the expression level of *KNGH1* is negatively associated with IAA concentration.

**Figure 5:**
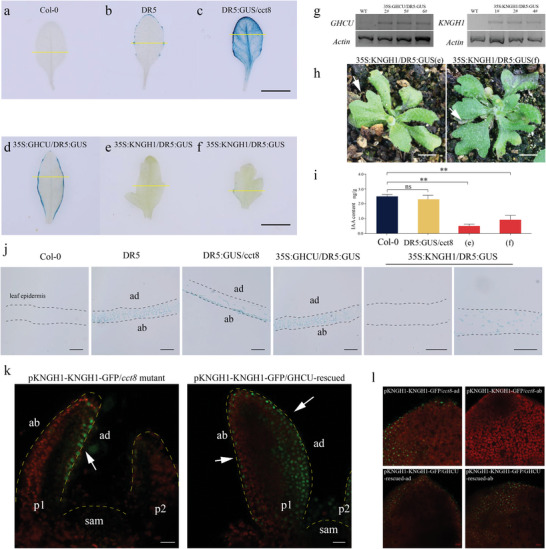
KNGH1 affects the distribution of auxin. GUS‐stained 7th true leaf of *Arabidopsis* a) Col‐0, b) DR5:GUS, c) DR5:GUS/*cct8*, d) 35S:*GHCU*/DR5:GUS, e,f) 35S:*KNGH1*/DR5:GUS. g) RT‐PCR was used to detect the expression of *GHCU* and *KNGH1* in (d), (e), and (f), *Actin* was used as an internal control. h) Images of abnormal leaves of (e) and (f) 35S:*KNGH1*/DR5:GUS. White arrows indicate abnormal leaves. i) IAA content of Col‐0, DR5:GUS/*cct8* and 35S:*KNGH1*/DR5:GUS. j) Transverse sections of (a–f). k) GHCU transfers KNGH1 from the adaxial to the abaxial side. Longitudinal section of 7‐day‐old *Arabidopsis* shoot apexes expressing KNGH1 (green), which is driven by *KNGH1* promoter, in *cct8* mutant and GHCU‐rescued line. The dotted yellow line indicates the central axis of the leaf. l) Surface view of 10‐day‐old *Arabidopsis* leaf adaxial‐abaxial epidermal cells. Scale bars = 0.5 cm in (h), 20 µm in (j), (k), and (l). Chlorophyll autofluorescence shows a red signal.

To determine the relationship between GHCU, KNGH1, and auxin response output, we developed *Arabidopsis* plants transformed with the reporter gene *GUS* under the control of the auxin‐responsive DR5 promoter (*At*DR5:GUS). This promoter has been widely used to visualize auxin response at the cellular level in *Arabidopsis* and maize.^[^ ^32^, ^33^ ^]^ We crossed DR5:GUS plants with the cup‐shaped leaf *Arabidopsis* mutant *cct8* (homolog of *GHCU*) to generate DR5:GUS/*cct8* plants. Subsequent GUS staining revealed auxin response signals to be concentrated on the leaf margin of DR5:GUS plants, but spread throughout the entire leaf in DR5:GUS/*cct8* plants (**Figure** **5**a–c). This indicates that loss of *CU* gene leads to total alteration of the distribution of auxin response output.

We further overexpressed that *GHCU is* driven by the 35S promoter in DR5:GUS plants to create 35S:GHCU/DR5:GUS plants. In these plants, we observed the auxin response signal to remain restricted to the leaf margin, similar to DR5:GUS plants (Figure 5d). This suggests that GHCU is necessary for regulating auxin response in the leaf, but excess GHCU does not affect auxin response. This might explain the normal leaves of *GHCU*‐overexpressing tobacco lines (Figure S8a,d, Supporting Information).

To investigate whether auxin response is associated with KNGH1, we ectopically expressed *KNGH1* in DR5:GUS *Arabidopsis*. The resulting plants (35S:KNGH1/DR5:GUS) exhibited severely‐lobed leaves (Figure 5e–g).^[^ ^34^, ^35^ ^]^ GUS staining showed the DR5 signal intensity to be very weak in 35S:KNGH1/DR5:GUS plants compared to DR5:GUS plants. Consistent with that result, 35S:KNGH1/DR5:GUS plants demonstrated a significant reduction of leaf IAA content, similar to the *KNGH1*‐overexpressing tobacco (Figure 5h,i), indicating that excessive KNGH1 inhibits auxin response.

To further examine the auxin response signal in the leaf adaxial‐abaxial boundary, we conducted detailed transverse section imaging. The GUS signal was found to be widely distributed in both the mesophyll cells and adaxial‐abaxial epidermis of 35S:CHCU/DR5:GUS and DR5:GUS *Arabidopsis* plants; conversely, it was specifically detected in the abaxial epidermis in the hybrid DR5:GUS/*cct8* plants. This suggests that normal GHCU is critical for auxin response on both the adaxial and abaxial sides. When KNGH1 was ectopically expressed, the GUS signal was either completely absent or present in very small amounts in the mesophyll (Figure 5j).

To investigate KNGH1 expression in leaves, we introduced *KNGH1* driven by its native promoter into the *Arabidopsis* cup‐shaped leaf mutant *cct8* and GHCU‐*cct8* rescued lines respectively. In five‐day‐old homozygous seedlings, the GFP signal was only detected in the adaxial epidermis and leaf tips of pKNGH1:KNGH1:GFP/*cct8* transgenic plants, but was observed in both the abaxial‐adaxial epidermis and mesophyll in pKNGH1:KNGH1:GFP/GHCU‐*cct8*‐rescued plants (Figure 5k), although GFP signal was much stronger in the adaxial side. In the young leaves of ten‐day‐old pKNGH1:KNGH1:GFP/GHCU‐*cct8*‐rescued plants, the adaxial and abaxial epidermis exhibited GFP signals of near similar strength, while in pKNGH1:KNGH1:GFP/*cct8* plants it was strictly limited to the adaxial epidermis (Figure 5l). These findings indicate that *KNGH1* expression is localized to the adaxial side and leaf tips, but KNGH1 protein could be transported from the adaxial to the abaxial epidermis with the help of GHCU, which functions as a molecular chaperone during leaf development. We further performed transient transformation of GHCU:RFP and KNGH1:GFP into *Nicotiana benthamiana* leaves. Strong fluorescence signals were observed in the nucleus and membrane; however, upon plasmolysis, no evidence of enrichment was found at the PD (Figure S11, Supporting Information), indicating that GHCU protein is not enriched at the PD. These results suggest that GHCU aids in KNGH1 movement from the adaxial to the abaxial boundary but not through the PD.

### 2.6 scRNA‐Seq and Spatiotemporal Transcriptomics Revealed Auxin Response and Synthesis to Determine Leaf Shape

**Figure 6:**
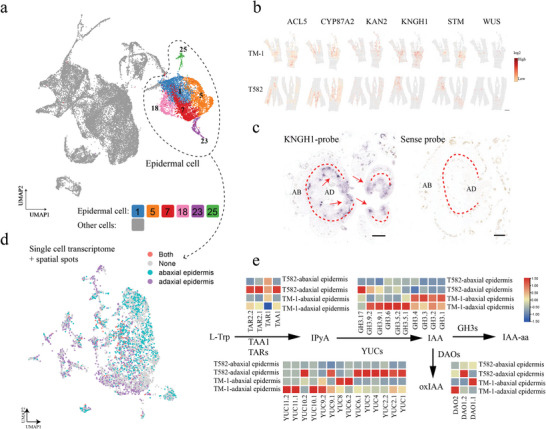
Cell heterogeneity within Allotetraploid Upland cotton shoot tips based on scRNA‐seq and spatial transcriptomics. a) UMAP visualization for the identification of 27 cell clusters from 27 212 cells in shoot tips. b) Illustrations of spatial expression distributions for marker genes. Scale bars = 1 mm. c) RNA in situ hybridization of KNGH1 gene. Scale bars = 100 µm. d) spatial transcriptomics allows separation between adaxial and abaxial epidermal cells. Distribution of upper and lower epidermal cells with spatial information. Cyan dots represent abaxial epidermal cells and purple dots represent adaxial epidermal cells. e) Spatial information of all key genes in the IAA synthesis pathway. DIOXYGENASE FOR AUXIN OXIDATION (DAO), GRETCHEN HAGEN3(GH3), indole‐3‐pyruvic acid (IPyA), YUCCA flavin‐containing monooxygenases (YUC), indole‐3‐acetic acid (IAA), tryptophan aminotransferase of Arabidopsis (TAA), tryptophan aminotransferase‐related protein (TAR).

The results mentioned above demonstrated that GHCU facilitates the trafficking of KNGH1, which leads to the difference in auxin response output between the adaxial and abaxial sides of a leaf. To better understand the transcriptional regulatory network responsible for adaxial‐abaxial patterning, we conducted a combination analysis of single‐cell RNA sequencing (scRNA‐seq) and spatiotemporal transcriptomic sequencing. Twenty‐seven distinct cell clusters were grouped into eight cell populations (**Figure** **6**a), namely epidermal cell and other cells (mesophyll cell, xylem cell, companion cell, phloem parenchyma cell, pigment gland cell, guard cell, and undefined cell).

Spatiotemporal transcriptomics were performed on TM‐1 and T582 shoot tip regions (Figure S12, Supporting Information). To validate the accuracy of the spatiotemporal transcriptomic data, we examined the spatial expression of several known genes. Among these, *ACAULIS5* (*ACL5*) showed predominant expression in xylem cells, while shoot meristemless (*STM*), *WUSCHEL* (*WUS*), and *KNGH1* exhibited primary expression in the shoot apical meristem (SAM) and young leaves. Additionally, *CYP87A2*, which encodes a protein with a cytochrome P450 domain, was found to be expressed in epidermal cells (Figure 6b). The observed spots from leaf, stem, and shoot apical meristem tissues were consistent with the corresponding histological examination, providing support for our classification. We confirmed the accuracy of the spatial transcriptome results through in situ hybridization, where the *KNGH1* gene showed high expression in the adaxial boundary of young cotton leaf transections in cotton (Figure 6c), consistent with its tissue location from promoter expression (Figure 5k,l).

Next, we focused our analysis on the epidermal cell group. By combining the single‐cell results with histological spatial information, we successfully distinguished four types of epidermal cells based on their association with either adaxial or abaxial sides: both, none, abaxial epidermis, and adaxial epidermis (Figure 6d; Figure S13, Supporting Information). We also created a heat map of reported abaxial‐adaxial marker genes to validate the cell clustering results along the proximal and distal axes (Figure S14, Supporting Information). Our results confirmed different auxin response signaling in the adaxial and abaxial boundaries of TM‐1 and T582. Next, we examined the expression of key genes in the IAA synthesis pathway. *YUCs* encode flavin‐containing monooxygenases that catalyze IPyA decarboxylation to IAA.^[^ ^36^, ^37^ ^]^ This is rate‐limiting and an irreversible step in auxin synthesis. Interestingly, we found *YUCs* to be highly expressed in both adaxial and abaxial sides of TM‐1 leaves, but only in the adaxial epidermis of T582 (Figure 6e). More than half (8/14) of *YUCs* showed high expression in T582 adaxial epidermis, while all *YUCs* exhibited little or no expression in T582 abaxial epidermis cells. In general, the results of the combination analysis support and validate the differences in IAA content with respect to the adaxial‐abaxial sides of leaves. Combined with our findings concerning KNGH1, we speculate that KNGH1 directly or indirectly regulates YUCs, which regulate the auxin response in the adaxial‐abaxial boundary of leaves.

**Figure 7:**
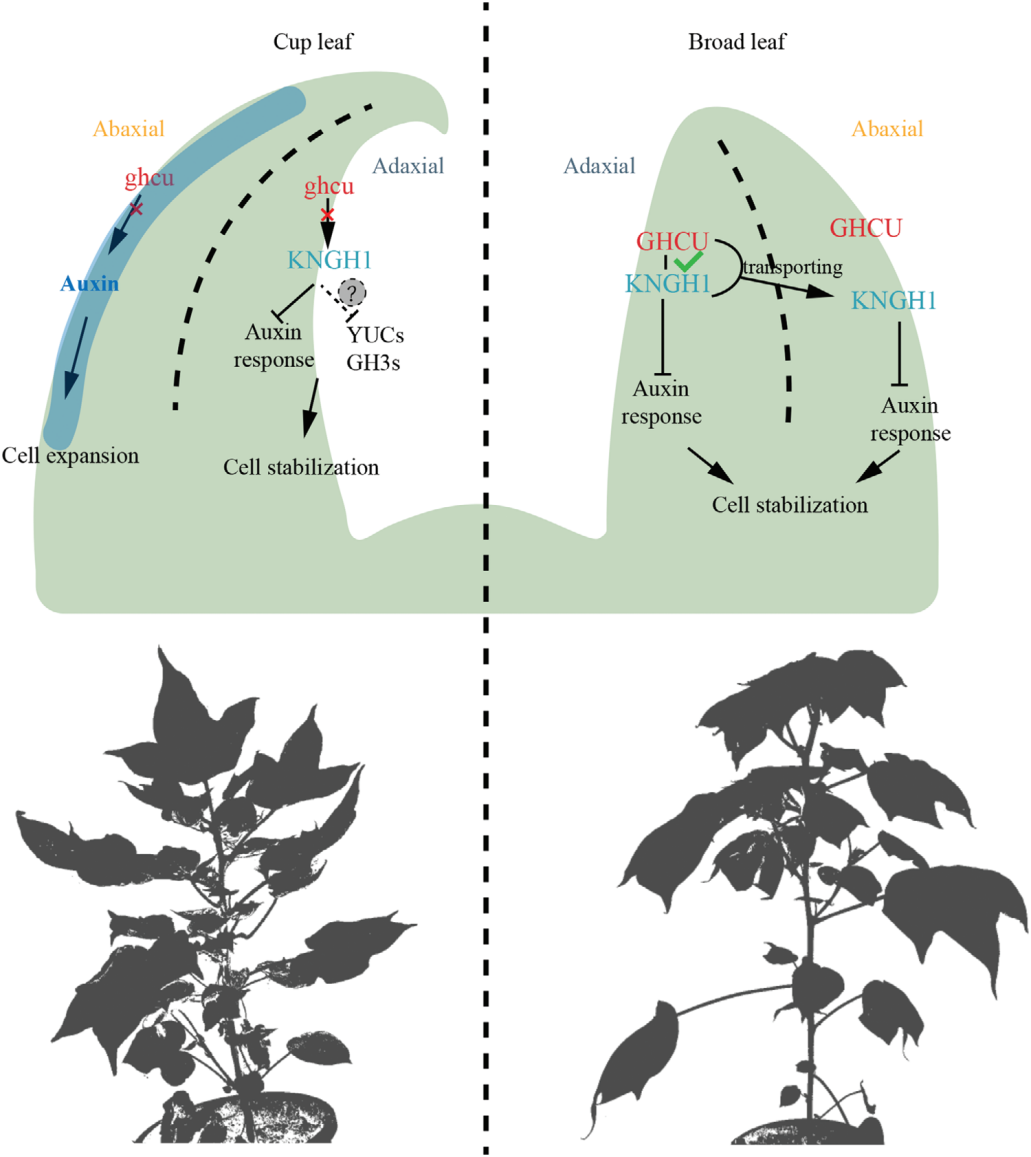
A working model for explaining leaf blade flattening. The polarity gene KNGH1 and auxin regulate leaf shape through cell proliferation.

Synthesizing these results, we propose a working model that explains leaf blade flattening (**Figure** **7**). As a molecular chaperone, GHCU is widely expressed throughout the leaf, while KNGH1 expression is restricted to the adaxial boundary. In wild‐type cotton, GHCU_TM‐1_ physically interacts with KNGH1 and transports it to the abaxial boundary cells. However, the mutant form ghcu_T582_ lacks the ability to transfer KNGH1, hence KNGH1 remains restricted to the adaxial boundary. As KNGH1 inhibits the auxin response, this leads to higher auxin content on the abaxial side compared to the adaxial side. This imbalance produces quicker cell proliferation on the abaxial side, inducing upward curling of the leaf blade.

## 3 Discussion

Mutants with cup‐shaped leaf morphology provide valuable models for studying leaf flattening and crop architecture breeding. Such mutants, regulated by phytohormones or mechanical structures, have been discovered in plants other than cotton, like *Brassica napus* and rice.^[^ ^38^, ^39^, ^40^ ^]^ Despite the cup‐shaped leaf phenotype being known in upland cotton as early as 1928,^[^ ^23^, ^41^ ^]^ the genes and mechanisms responsible for this trait remain unknown. Our research has revealed that the formation of a cup‐shaped leaf pattern in upland cotton is attributable to the larger size and disordered arrangement of epidermal cells on the lower side of the leaf. We found that GHCU, a molecular chaperone, is present throughout the entire leaf, whereas KNGH1 is restricted to the upper side of the leaf (Figures 5k and 6c). In wild‐type cotton (TM‐1), GHCU physically interacts with KNGH1 and transports it to the abaxial epidermis cells. However, the mutant form ghcu_T582_ lacks the ability to interact with and transfer KNGH1. As a result, KNGH1 is distributed and inhibits auxin response only in the adaxial epidermis of T582 leaves, leading to a relatively more active auxin response and biosynthesis on the abaxial side. This imbalance induces upward curling of the leaf blade. Based on these findings, we propose a working model to explain the curling leaf in cotton. During the early stages of leaf development, a newly emerging leaf primordium typically exhibits a relatively small adaxial domain and a more developed and enlarged abaxial domain. Cell proliferation and growth in the adaxial domain are lower.^[^ ^42^, ^43^ ^]^ However, cell proliferation in the adaxial domain must eventually surpass that in the abaxial domain so as to achieve a final balance between the two sides of the leaf. Transport of KNGH1 in young leaves of T582 plants is limited to the adaxial side; this limitation leads to a decrease in adaxial auxin biosynthesis and cell growth, which ultimately results in the formation of cup‐shaped leaves. Meanwhile, the leaves of the plants overexpressing *KNGH1* appeared shrunken, but not curling, compared to control plants (Figure S10, Supporting Information), and also showed significantly decreased IAA content, entirely due to the abundant expression of KNGH1. Maintaining a balance of auxin levels in both the adaxial and abaxial epidermis is crucial for achieving flattened leaf blades.

*GHCU* encodes a chaperonin subunit of the chaperonin containing TCP‐1 (CCT) protein, which is conserved in various plant species, including tobacco, *Arabidopsis*, and maize. CCT is a general chaperonin and is the only chaperonin found in the cytosol of all eukaryotes. CCT proteins are ATPases that assemble into single‐ and double‐ring protein machines, capable of binding and sequestering non‐native proteins in their central cavities. They can assist in folding these proteins to their native states, driven by ring cycle(s) of ATP binding and hydrolysis.^[^ ^44^ ^]^ The CCT family consists of nine members, some of which have been linked to human diseases.^[^ ^45^, ^46^, ^47^ ^]^ Each subunit of CCT contains an equatorial domain with the ATP‐binding site,^[^ ^48^ ^]^ and extensive molecular studies have identified that the eight CCT subunits occupy fixed positions in the chaperonin ring. Our experimental results, as shown in Table S4 (Supporting Information), indicate that CCT2 and GHCU may be aligned in a chaperonin ring, as CCT2 was included in the list of screened genes. Research in *S. cerevisiae* indicates individual CCT subunits have various functions within cells,^[^ ^49^ ^]^ but only limited studies have been conducted in plants. Silencing *CCT5* was found to disrupt the movement of ribonucleoprotein complexes from hairy roots to new leaves via the phloem,^[^ ^50^ ^]^ indicating that chaperonins are essential for cell‐to‐cell trafficking of a subset of mobile transcription factors. However, it is currently unknown whether the CCT protein GHCU operates as an individual unit or forms part of a chaperonin ring.

The variant of GHCU identified in this study (G to T, causing an amino acid substitution from Glu^518^ to Asp^518^) influences its bonding with KNGH1. *KNGH1* is a homolog of the *Arabidopsis* gene *KNAT1* (also known as *BREVIPEDICELLUS*), which encodes an HD protein that is normally expressed in the shoot apical meristem. Ectopic expression of *KNAT1* in leaves alters leaf morphology. In *Arabidopsis*, a high level of auxin antagonizes *KNOX1* expression for organ initiation in the peripheral zone of the meristem.^[^ ^29^, ^35^ ^]^ Previous studies in *KNAT1* mutant plants have reported more severe effects on cell differentiation, elongation, and growth on the abaxial side compared to the adaxial side, resulting in changes in pedicel growth angles.^[^ ^51^ ^]^ Our findings are consistent with this, as we observed that the interaction of GHCU with KNGH1 promotes the growth of epidermis cells on the abaxial side. Since both leaf and flower primordia differentiate from the SAM, we hypothesize that KNAT1 regulates epidermal cell development on the abaxial‐adaxial side in a similar manner. Additionally, our data revealed that KNGH1 regulates the leaf response to auxin, particularly in the abaxial‐adaxial epidermis, with KNGH1 and auxin mutually inhibiting each other in the leaf epidermis. Leaves overexpressing *KNGH1* exhibited changes in leaf shape alongside minimal auxin response activity (Figure 5h,i). Therefore, we identified KNGH1 as a novel auxin response inhibitor that affects auxin response in the cotton leaf.

During leaf development, an auxin maximum is first formed at the tip of the young leaf primordia, which is thought to direct distal growth.^[^ ^52^ ^]^ The crimping of the cup‐shaped leaves of T582 is most noticeable at the tips of young leaves, providing further evidence for the hypothesis that auxin is directly related to the cup‐shaped phenotype. Recent studies on *YUC* genes have provided more direct evidence that auxin plays a key role in the promotion of leaf development and leaf margin formation.^[^ ^53^, ^54^, ^55^ ^]^ YUC proteins, which control the rate‐limiting step in Trp‐dependent auxin biosynthesis, are crucial for local auxin biosynthesis and have important roles in leaf margin development.^[^ ^53^ ^]^ However, in *Arabidopsis*, *YUC* genes are up‐regulated in ectopic adaxial and abaxial juxtaposition leaves.^[^ ^56^ ^]^ In rice, loss of YUC activity due to mutation of *narrow leaf 7* (*nal7*) also results in a reduction of endogenous auxin content and a narrow leaf phenotype.^[^ ^57^ ^]^ Thus, *YUC* genes are expressed in response to adaxial‐abaxial juxtaposition, and local auxin synthesis by *YUC* is partly responsible for adaxial‐abaxial development in plants. In this study, we used scRNA‐seq and spatiotemporal transcriptomic integrative analysis to demonstrate that *YUCs* are expressed in varying abundance in adaxial‐abaxial epidermal cells of the two cultivars (Figure 6e).

The similarity observed between the *Arabidopsis cct8* mutant and *GHCU*‐edited plants indicates a conserved regulatory pathway for the development of adaxial‐abaxial epidermis in both *Arabidopsis* and cotton. Overall, our findings provide evidence that *GHCU* plays a crucial role in regulating the development of epidermal cells, specifically with regard to leaf shape. Through our study, we have uncovered the molecular connection between GHCU and the mechanisms responsible for curly leaf formation. This knowledge can be valuable in the precise breeding of cotton varieties with desirable and productive plant architecture.

## 4 Experimental Section

### Plant Materials

Texas 582 (T582) is a multiple‐recessive marker line of upland cotton (*Gossypium hirsutum*) with the same genetic background as the reference line TM‐1.^[^ ^58^ ^]^ T582 exhibits five mutant phenotypes including the cup leaf phenotype, which is controlled by a recessive gene locus, *cu*. All cotton plants used in this study (Yu668, *GHCU* gene‐edited cotton lines, TM‐1, and T582) were cultivated in the field at the experimental station of Zhejiang University (ZJU) in China. The cross between TM‐1 and T582 was performed at the Jiangpu Breeding Station, Nanjing Agricultural University (JBS/NAU), resulting in the development of an F_2_ mapping population (2200 individuals) and a BC_1_ population (419 individuals).^[^ ^26^ ^]^ Leaf primordia were carefully removed, immediately snap‐frozen in liquid nitrogen, and stored at −70 °C for DNA and RNA extraction.

All *Arabidopsis* seed stocks are on the Col‐0 background unless otherwise stated. The pKNGH1:KNGH1:GFP construct was generated by cloning 2 kb of the *KNGH1* promoter, located upstream of the *KNGH1* coding sequence, from upland cotton. The *Arabidopsis* pKNGH1:KNGH1:*GFP*/*cct8* cross was produced by crossing plants that were positive for pKNGH1:KNGH1:*GFP* with the cup‐shaped leaf mutant *cct8*. The GHCU*‐cct8*‐rescued line was generated by ectopic expression of *GHCU* in *cct8* mutant *Arabidopsis*. The pKNGH1:KNGH1:GFP/GHCU*‐cct8*‐rescued line was generated through crossing.

### qRT‐PCR Analysis

Total RNA was reverse‐transcribed to cDNA using a HiScript II Reverse Transcriptase Kit (Vazyme, Nanjing, China). qRT‐PCR was conducted on an Applied Biosystems 7500 Fast Real‐Time PCR System (Life Technologies, Carlsbad, CA, USA) in a 20 µL volume containing 100 ng of cDNA, 4 pm of each primer, and 10 µL of AceQ qPCR SYBR Green Master Mix (Vazyme) according to the manufacturer's protocol. The PCR conditions were as follows: primary denaturation at 95 °C for 20 s, followed by 40 amplification cycles of 3 s at 95 °C and 30 s at 60 °C. Melting curve analysis was performed to ensure no primer dimer formation. Data were evaluated using the comparative cycle threshold method described by Livak and Schmittgen.^[^ ^59^ ^]^ Three biological replicates (three samples harvested from three plants, one from each) were performed per reaction, each with three technical replicates (from the same sample). Mean values and standard errors were calculated based on data from three replicates.

### Virus‐Induced Gene Silencing (VIGS) Assay

For the VIGS assay, a 313‐bp fragment of *GHCU*, corresponding to bases 1503–1653 of the *GHCU* gene and 163 bp of its 3′UTR, was amplified by PCR. The resulting PCR product was cloned into the pTRV2 vector, producing a construct designated as pTRV2‐*GHCU*. *Agrobacterium* cells respectively carrying pTRV1 and pTRV2‐*GHCU* were re‐suspended in an infiltration medium (10 mm MgCl_2_, 10 mm MES, 200 µM acetosyringone), and adjusted to an OD600 of 2.0. The suspensions were mixed at a 1:1 ratio. The resulting mixture was then infiltrated into the cotyledons of 10‐day‐old seedlings. After infiltration, the seedlings were placed in the dark for 24 h and then incubated at 23 °C with a 16‐h light/8‐h dark cycle. TM‐1 cotton plants served as the receptor in the VIGS assay for silencing of *GHCU*. Plants transformed with an empty vector (TRV:00) were used as experimental controls. The chloroplast alterados 1 (*CLA1*) gene was used as an indicator of the silencing effect.^[^ ^60^ ^]^ Thirty seedlings were included in each experimental group. Photos were taken 3 weeks after infiltration, and leaves were collected for expression analysis.

### Cotton Transformation

The vector pRGEB32‐GhU6.9‐NPT II was constructed for stable genetic transformation to knock out *GHCU* in cotton.^[^ ^61^ ^]^ This vector was introduced into *G. hirsutum* Yu668 via *Agrobacterium* tumefaciens‐mediated transformation.^[^ ^62^ ^]^ *Agrobacterium* strain LBA4404 holding the CRISPR/Cas9 plasmid vector was grown in Luria–Bertani liquid medium supplemented with 50 mg L^−1^ kanamycin and 10 mg L^−1^ rifampicin at 28 °C for 24 h. The bacteria were resuspended in a liquid MSB medium and adjusted to an OD600 of 0.3–0.5.

### LUC Complementation Imaging (LCI) Assays

For LCI assays, the sequences of *GHCU_TM‐1_* and *ghcu_T582_* were individually ligated with the N‐terminal fragment of luciferase (nLUC) to form *GHCU*‐nLUC. The full‐length coding sequence of *KNGH1* was fused with the C‐terminal fragment of luciferase (cLUC). FT1 and FD were used as positive controls.^[^ ^63^ ^]^ Images were captured using a low‐light, cooled, charge‐coupled device imaging system (Tanon, Fremont, CA, USA).^[^ ^64^ ^]^

### Co‐Immunoprecipitation (co‐IP) Assays

Co‐IP was conducted as previously described.^[^ ^65^ ^]^ Briefly, KNGH1‐RFP and GHCU‐GFP or vector‐GFP and vector‐RFP were transiently co‐expressed in *N. benthamiana* leaves for 3 days. Lysates were incubated with anti‐GFP or anti‐RFP affinity M2 beads (Sigma–Aldrich, St. Louis, MO, USA) at 4 °C for 2 h. The beads were washed three times with PBS, and the immunoprecipitated proteins were examined by immunoblotting.

### Yeast Two‐Hybrid (Y2H) Assays

Y2H assays were conducted using the GAL4 vector system (Clontech, USA). *GHCU* and *KNGH1* were respectively cloned into the pGBKT7 and pGADT7 vectors, whose constructs were then co‐transformed into the yeast strain Y2H. Transformed cells were adjusted to an OD600 of 0.4–0.6 and grown on SD/‐Trp‐Leu or SD/‐Trp‐Leu‐His‐Ade plates for 3–7 days at 30 °C.

### ESI‐HPLC‐MS/MS

Auxin was purchased from Sigma. Tissues were snap‐frozen in liquid nitrogen and immediately ground to powder. The extraction procedure was repeated twice after adding 4 µL of the internal standard. The combined extract was then concentrated under reduced pressure and mixed with 35 mg Sep‐Pak Plus C18 Cartridge (Waters). Following solid phase extraction, each well was dried under nitrogen for 25 min. Four microliters of the solution were analyzed using an LC‐ESI‐MS/MS system, which consisted of an Agilent 1260 HPLC system coupled to an API6500 triple‐quadrupole‐stage mass spectrometer (Applied Biosystems/MDS Sciex), operated in multiple reaction monitoring mode (Nanjing Convinced‐test Technology Co., Ltd, Nanjing, China).

### Promoter Analysis

The 2.0 kb fragment upstream of the *GHCU* transcriptional start site was cloned and inserted into the pCAMBIA1391 vector to construct ProGHCU‐GUS, in which GUS expression is driven by the *GHCU* promoter. This construct was introduced into *N. benthamiana* and *Arabidopsis* via *Agrobacterium*‐mediated transformation.

### RNA‐ Seq Data Processing

Single‐cell RNA‐seq data processing was performed using the Cell Ranger 4.0 pipeline with the recommended default parameters. The Illumina sequencing output generated FASTQs, which were aligned to the cotton genome (ZJUV2.1) using the STAR algorithm.^[^ ^66^ ^]^ Gene‐Barcode matrices were generated for each sample by counting Unique Molecular Identifiers (UMIs) and filtering out non‐cell associated barcodes. Quality control and downstream analysis of the resulting matrices were performed using the Seurat (v3.2.0) R toolkit.^[^ ^67^ ^]^ Default parameters were used for all functions, unless specified otherwise. To exclude low‐quality cells, we applied a standard panel of three quality criteria: 1) number of detected transcripts; 2) number of detected genes; and 3) percent of reads mapping to mitochondrial genes (quartile threshold screening criteria). Expression of mitochondria genes was calculated using the PercentageFeatureSet function of the *seurat* package. To extract a subset of variable genes, normalized data was analyzed while controlling for the strong relationship between variability and average expression. Next, data were integrated from different samples after identifying “anchors” between datasets using FindIntegrationAnchors and IntegrateData in the *seurat* package.^[^ ^68^ ^]^ Then, principal component analysis (PCA) was performed and the data were reduced to the top 30 PCA components after scaling. The clusters were visualized on a 2D map produced with t‐distributed stochastic neighbor embedding (t‐SNE).^[^ ^69^ ^]^

Graph‐based clustering of cells was performed using the PCA‐reduced data with the Louvain Method ^[^ ^70^ ^]^ after computing a shared nearest neighbor graph. For sub‐clustering, the same procedure of scaling, dimensionality reduction, and clustering to the relevant data subset (usually restricted to one type of cell) was applied. The Wilcoxon rank‐sum test was used to find significant differentially expressed genes in each cluster compared to the remaining clusters. SCINA ^[^ ^71^ ^]^ and known marker genes were used to identify cell types. Epidermal‐specific genes such as 3‐KETOACYL‐COA SYNTHASE (KCS)∖FIDDLEHEAD(FDH) to be dominantly expressed in the EC population (≈22%; clusters 1, 5, 7, 18, 23, and 25) (36, 37) (Figure S15, Supporting Information) were found.

For 10× Visium spatial sequencing, raw FASTQ files and histology images were processed by sample with the Space Ranger software v. 1.2.2, which uses STAR ^[^ ^72^ ^]^ for genome alignment. The per‐spot quality metrics was evaluated using the SeuratV4.0 R Bioconductor package. UMIs with fewer than 200 detected spots were excluded.

Gene expression from each voxel was normalized by means of sctransform ^[^ ^73^ ^]^ in Seurat, which uses regularized negative binomial models to account for technical artifacts while preserving biological variance. Then top 30 principal components were calculated and used to construct the KNN graph. Voxel clustering was performed using the Louvain algorithm. The clusters were visualized on a 2D map produced with t‐distributed stochastic neighbor embedding (t‐SNE) and Uniform Manifold Approximation and Projection (UMAP).^[^ ^74^ ^]^ SingleR was used to identify cell type. The Wilcoxon rank‐sum test was used to identify significant differentially expressed genes among each cluster compared to the remaining clusters.

### Integration of Single‐Cell RNA Sequencing Data into a Spatial Context

The upper and lower epidermal areas were separately delineated on H&E‐stained slices of two blank control samples and differentially expressed genes (DEGs) between the sides were identified. The spots of adaxial–abaxial are shown in Figure S13 (Supporting Information). DEGs were identified using the FindMarkers function was used with default parameters (min.pct = 0.1, logfc. threshold = 0.25); those with *p*‐value < = 0.005 were selected as significant DEGs. Taking the union of DEGs identified in both samples resulted in a final total of 579 genes. Based on logfc, these DEGs were then divided according to the side they were upregulated in: leaf‐ab (240 genes) or leaf‐ad (339 genes).

Generally, highly variable genes (HVGs) are used for dimensionality reduction. Rather than use HVGs, subclustering of epidermal cell populations in single‐transcriptome data based on the 579 DEGs obtained from the blank control was performed. The resolution was set at 0.5. Cell types were determined by gene set scoring analysis, in which each cell was scored using the AddModuleScore function. The upper quartile was taken as the threshold to determine whether a score was Positive or Negative. Cells with a Positive score in leaf‐ab were identified as belonging to the abaxial epidermis, and similarly for leaf‐ad with the adaxial epidermis. If both scores were Positive, the cell was labeled as “both”, and if both were Negative, the cell was labeled as “none”. For intergroup differences, the FindMarkers function with parameters set to min.pct = 0.1 and logfc.threshold = 0.25 were applied. Genes with *p*‐value adj ≤ 0.05 were considered significantly differentially expressed.

### Quantification and Statistical Analysis

The two‐tailed paired Student's *t*‐test was used to determine the significant difference between the two samples. Error bars in the figures represent the standard deviation of the mean, and “n” in figure legends denotes sample size. Asterisks (∗) indicate statistical significance at *p* < 0.05, and double asterisks (^∗∗^) at *p* < 0.01. At least three biological replicates were included in the analyses. Statistical analysis was performed using GraphPad Prism 6.0.

## Conflict of Interest

The authors declare no conflicts of interest.

## Author Contributions

Y.Z. and C.X. contributed equally to this work. T.Z. and Y.H. conceptualized and coordinated the project. Y.Z., C.X., and Z.S. conducted mapping. Y.Z., C.X., L.Y., and L.M. conducted cloning and validation. Y.Z. and Y.H. wrote and revised the manuscript. All authors discussed the results and commented on the manuscript.

## Supporting information

Supporting Information

## Data Availability

The data that support the findings of this study are available in the supplementary material of this article.
