# Supplementary material for: GHCU, a Molecular Chaperone, Regulates Leaf Curling by Modulating the Distribution of KNGH1 in Cotton
Source: Adv Sci (Weinh). 2024 Apr 26;11(26):2402816. doi: 10.1002/advs.202402816 (PMC11234424; doi:10.1002/advs.202402816)
Supplement: Supplementary file 1 — Supporting Information [file ADVS-11-2402816-s001.pdf]

ADVANCED SCIENCE
Open Access

Supporting Information

for *Adv. Sci.*, DOI 10.1002/advs.202402816

GHCU, a Molecular Chaperone, Regulates Leaf Curling by Modulating the Distribution of KNGH1 in Cotton

Yihao Zang, Chenyu Xu, Lishan Yu, Longen Ma, Lisha Xuan, Sunyi Yan, Yayao Zhang, Yiwen Cao, Xiaoran Li, Zhanfeng Si, Jieqiong Deng, Tianzhen Zhang and Yan Hu**

Supporting Information for

GHCU, a molecular chaperone, regulates leaf curling by modulating the function of KNGH1 in cotton

Yihao Zang, Chenyu Xu, Lishan Yu, Longen Ma, Lisha Xuan, Sunyi Yan, Yayao Zhang, Yiwen Cao, Xiaoran Li, Zhanfeng Si, Jieqiong Deng, Tianzhen Zhang and Yan Hu

#Correspondence and requests for materials should be addressed to Yan Hu (0016211@zju.edu.cn) and Tianzhen Zhang (cotton@zju.edu.cn)

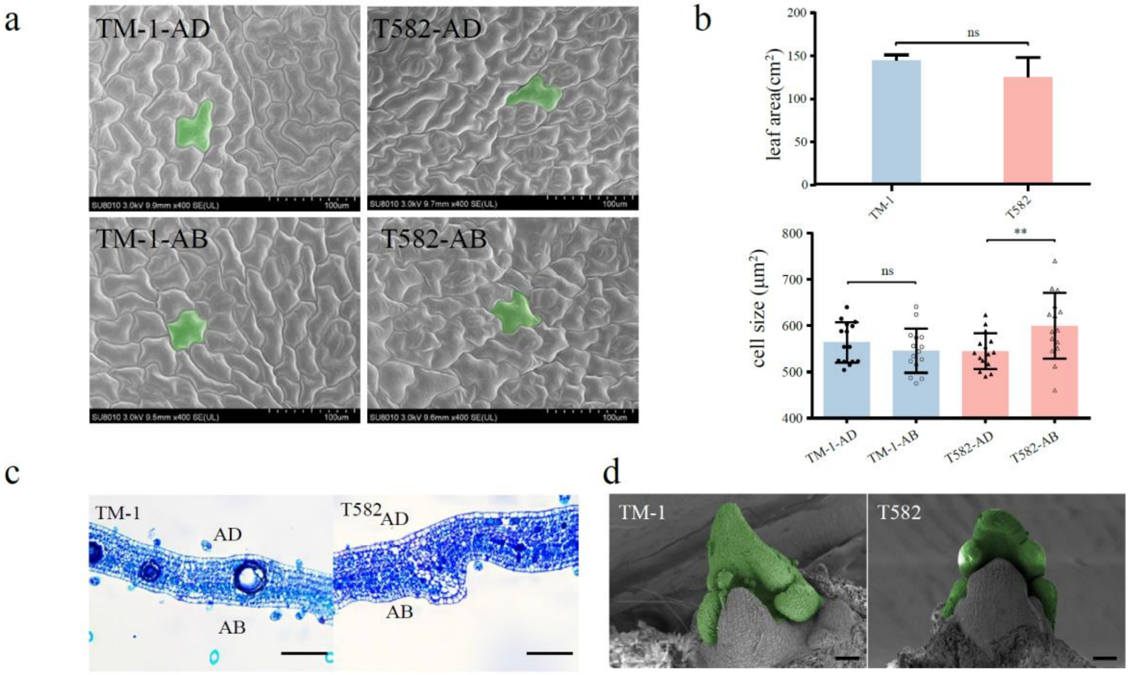

Fig. S1 Phenotypes of TM-1 and T582. (a) SEM images depicting leaf surfaces of parent plants. A single epidermal cell near the center is highlighted in green. (b) Statistical analysis of leaf area and cell size in TM-1 and T582. (c) Toluidine blue staining of paraffin sections using leaves from the parents. (d) SEM images showing the leaf primordium. The first true leaf of each parent is marked in green. AD refers to the adaxial side of the leaf, while AB refers to the abaxial side. Scale bars = 100 μm. *P*-values were determined using Student's t-test (**$p < 0.01$).

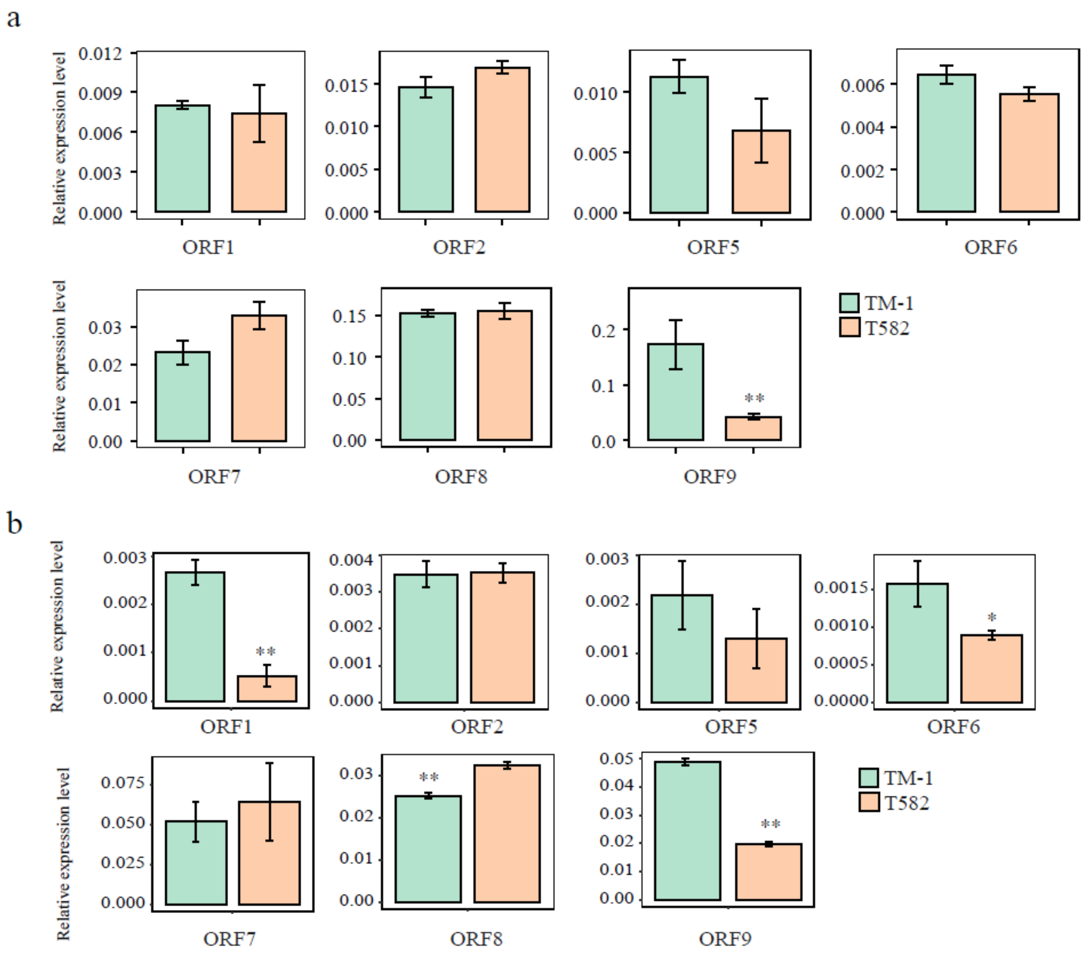

Fig. S2 Relative expression level of the seven selected candidate genes were analyzed by qRT-PCR in (a) leaf margin and (b) leaf primordium. The data is presented as the mean (±SD) of three experimental replicates; *P*-values were determined using Student's t-test (**p* < 0.05, ***p* < 0.01)

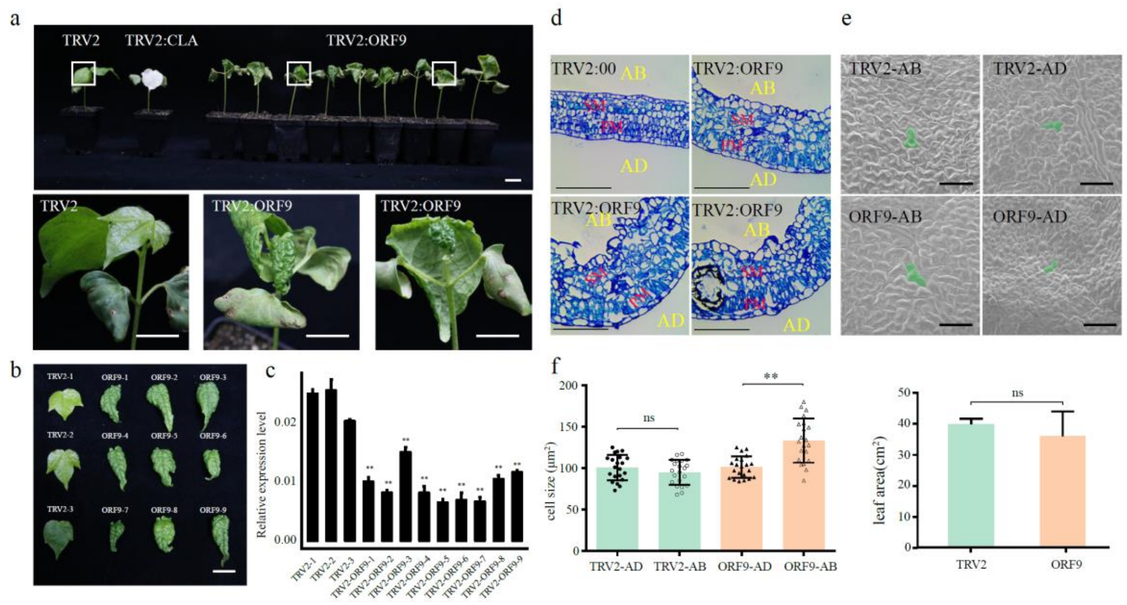

Fig. S3 RNA interference suppression of ORF9 by VIGS leads to abnormal cell morphology of the abaxial epidermis. (a) Suppression of ORF9 by VIGS leads to crumpled leaves. Three weeks after treatment, plants transfected with empty vector (TRV2:00) and RNAi (TRV2:ORF9) were observed. The lower panel magnifies the white boxed region in the upper panel. (b) Top view of the 2nd true leaf. (c) Relative expression of ORF9 in VIGS-treated cotton. (d) Toluidine blue staining of paraffin sections of TRV2:00 and TRV2:ORF9 plants. AD: adaxial, AB: abaxial, PM: Palisade mesophyll cells, SM: Spongy mesophyll cells. (e) SEM images of the adaxial-abaxial epidermis in (d). One epidermal cell near the center is highlighted in green. (f) Statistics of leaf area and cell size in (e). The data shown represent the mean (±SD) of three experimental replicates with n > 20. Scale bars = 5 cm in (a) and (b), 100 μm in (d) and (e). The *p*-values were determined using Student's t-test (**$p <$ 0.01).

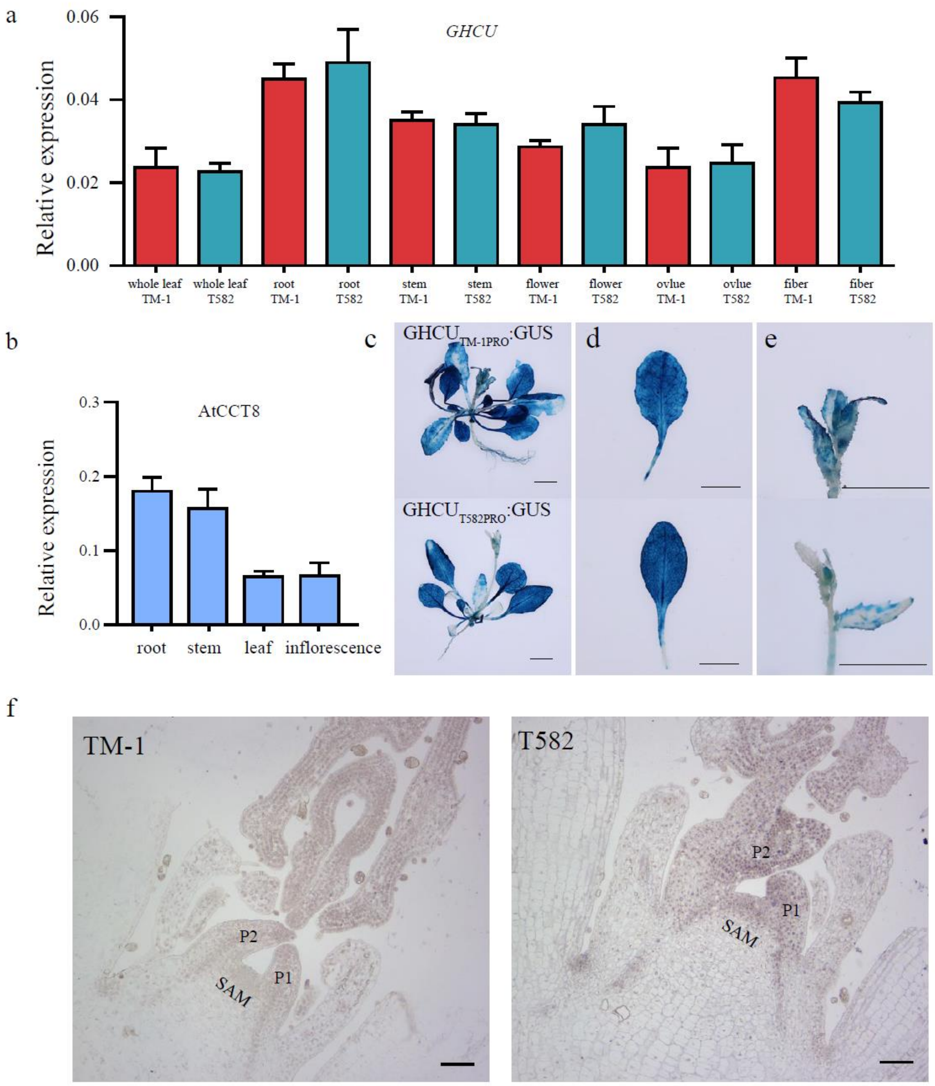

Fig. S4 Spatial expression pattern of *GHCU*. (a) Analysis of *GHCU* expression level in different organs of the parents by qRT-PCR. The error bars in the graph represent SD of three biological replicates. (B)The expression level of *AtCCT8* in *Arabidopsis* by qRT-PCR (c) GUS-stained whole plants of 6-week-old *Arabidopsis*. Magnification of the GUS-stained 5th true leaf (d) and inflorescence stem with flowers (e) of *Arabidopsis*. (f) Accumulation of *GHCU* transcripts, as visualized by RNA in situ hybridization of the SAM tissues of the parents. Primordium 1 (P1), Primordium 2 (P2), shoot apical meristem (SAM). Scale bars = 1 cm in (c), (d), and (e), and 100μm in (f).

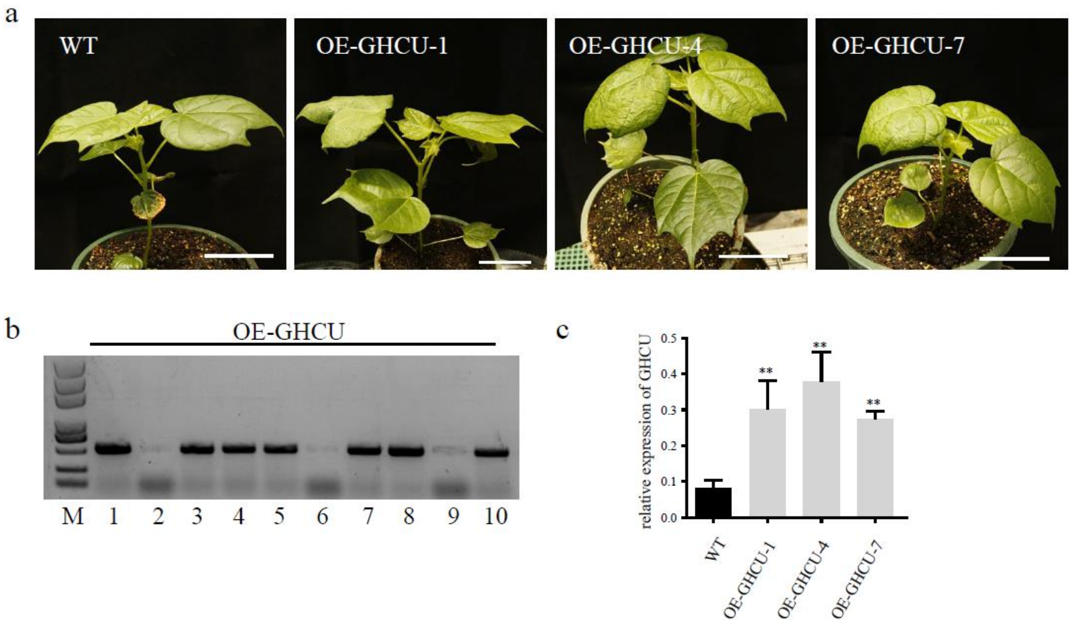

Fig. S5 Phenotypic analysis of *GHCU* overexpression in transgenic *Gossypium hirsutum*. (a) Photographs of three *GHCU*-overexpressing transgenic lines. (b) Identification of the target fragment in ten overexpression lines by PCR. (c) qRT-PCR analysis of three *GHCU*-overexpressing transgenic lines from (a). M, marker; OE, overexpression. Scale bars = 5 cm. *P*-values were determined using Student's t-test (**p < 0.01).

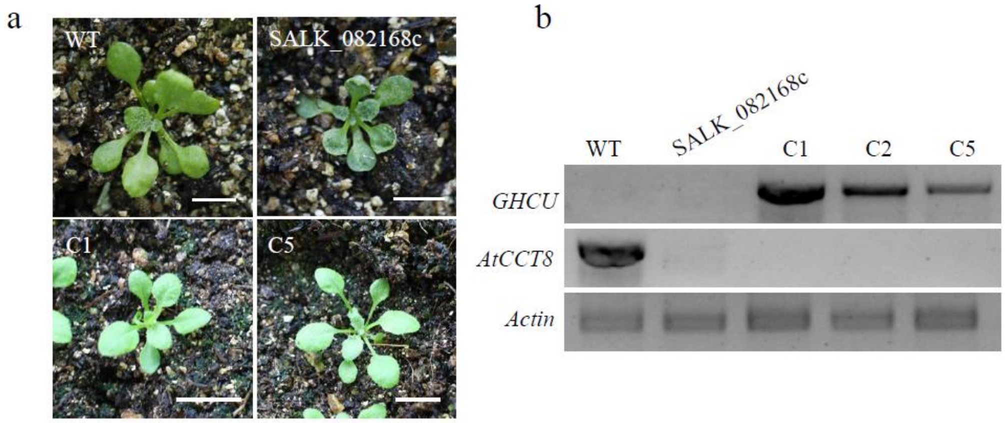

Fig. S6 Complementation of the *Arabidopsis* cct8 mutant through ectopic expression of *GHCU*. (a) The phenotypes of wild-type (WT) *Arabidopsis*, *cct8* (*SALK_082168c*), and *cct8* trans-formed with *GHCU* (C1 and C5). Scale bar = 0.5 cm. (b) The mRNA expression in WT, *cct8* and the complementation, is analyzed using RT-PCR. Actin was used as the internal control.

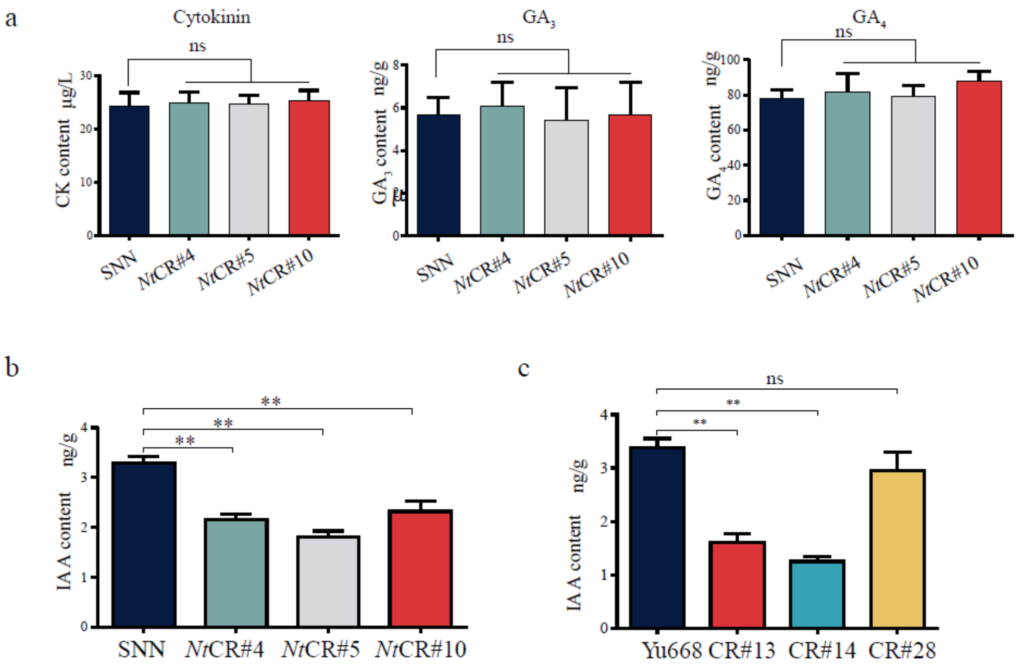

Fig. S7 Plant hormone content in tobacco and cotton using ESI-HPLC-MS/MS. (a) cytokinin (CK) and gibberellic acid$_{3/4}$ ($GA_{3/4}$) content in leaves from wild type SNN and three transgenic tobacco lines. (b) IAA content in leaves of three transgenic tobacco lines. (c) IAA content in leaves of three transgenic cotton lines. The *p*-values were determined using Student's t-test (**$p < 0.01$).

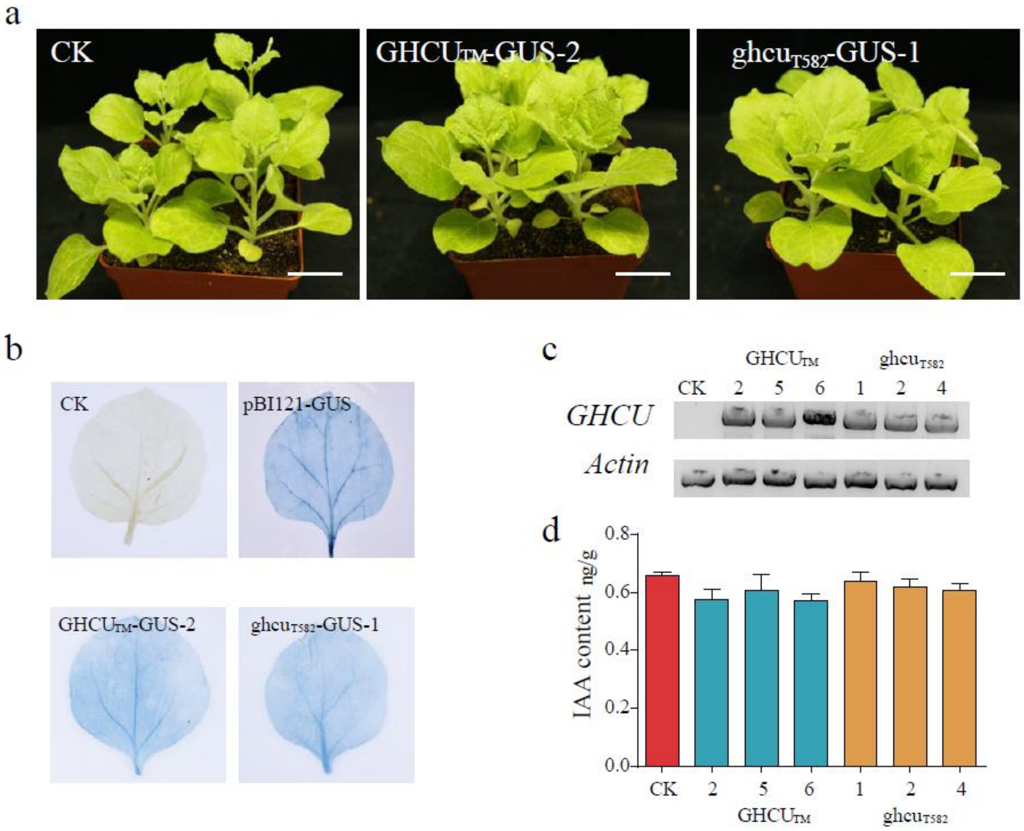

Fig. S8 Ectopic expression of *GHCU* in *Nicotiana benthamiana*. (a) Photographs of tobacco plants overexpressing GHCUTM/T582 are shown. (b) GUS-stained intact leaves of transgenic tobacco. are presented, with pBI121-GUS is serving as the positive control. (c) mRNA expression of CK, GHCUTM-1, and GHCUT582, was analyzed using RT-PCR., with *Actin* was utilized as an internal control. (d) The IAA content in the leaves of the plants in (a)shown in (a) was measured. Scale bars = 5 cm.

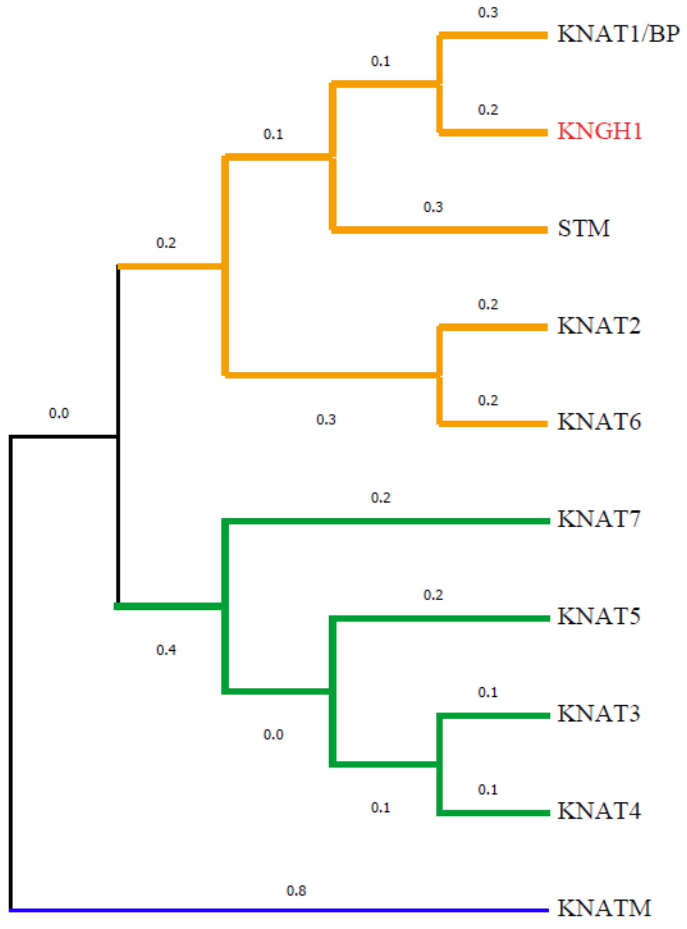

Fig. S9 Phylogenetic tree of three chrysanthemum KNOX proteins in *Arabidopsis* and KNGH1 in cotton. The yellow line represents the KNOX class I branch, the green line represents the KNOX class II branch, and the blue line represents the KNATM branch.

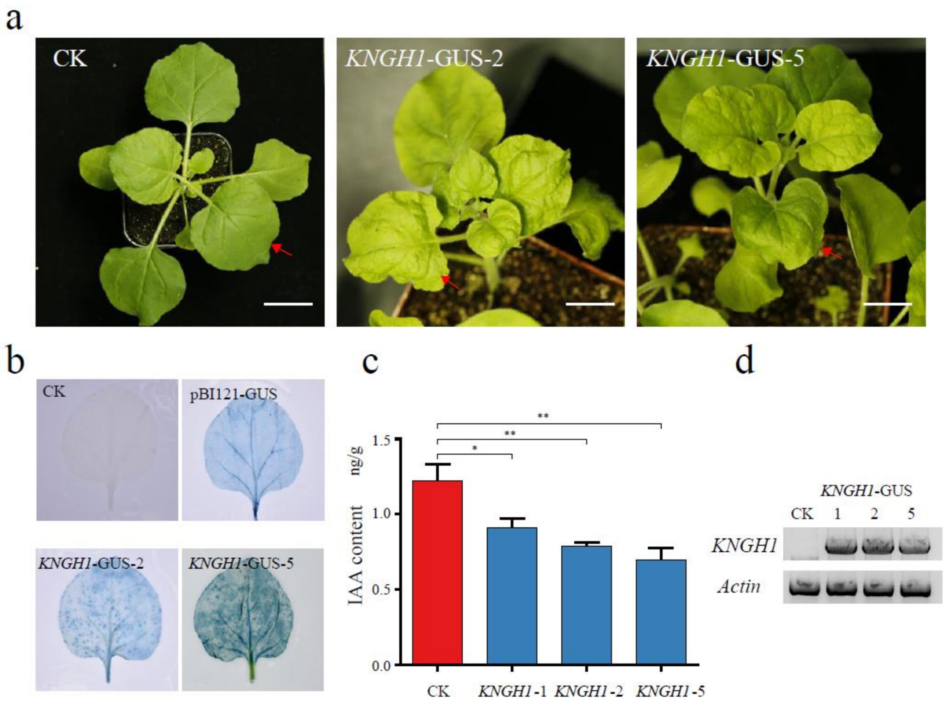

Fig. S10 Ectopic expression of *KNGH1* in *Nicotiana benthamiana*. (a) Photographs of tobacco plants overexpressing *KNGH1*. Abnormal leaves are marked with red arrows. (b) GUS-stained whole leaves of transgenic tobacco. pBI121-GUS was the positive control. (c) The IAA content in the leaves of the plants in (a). (c) mRNA expression of CK and *KNGH1*-GUS was analyzed using RT-PCR. Actin was used as an internal control. Scale bars = 5 cm. *P*-values were determined by Student's t-test (***p* < 0.01).

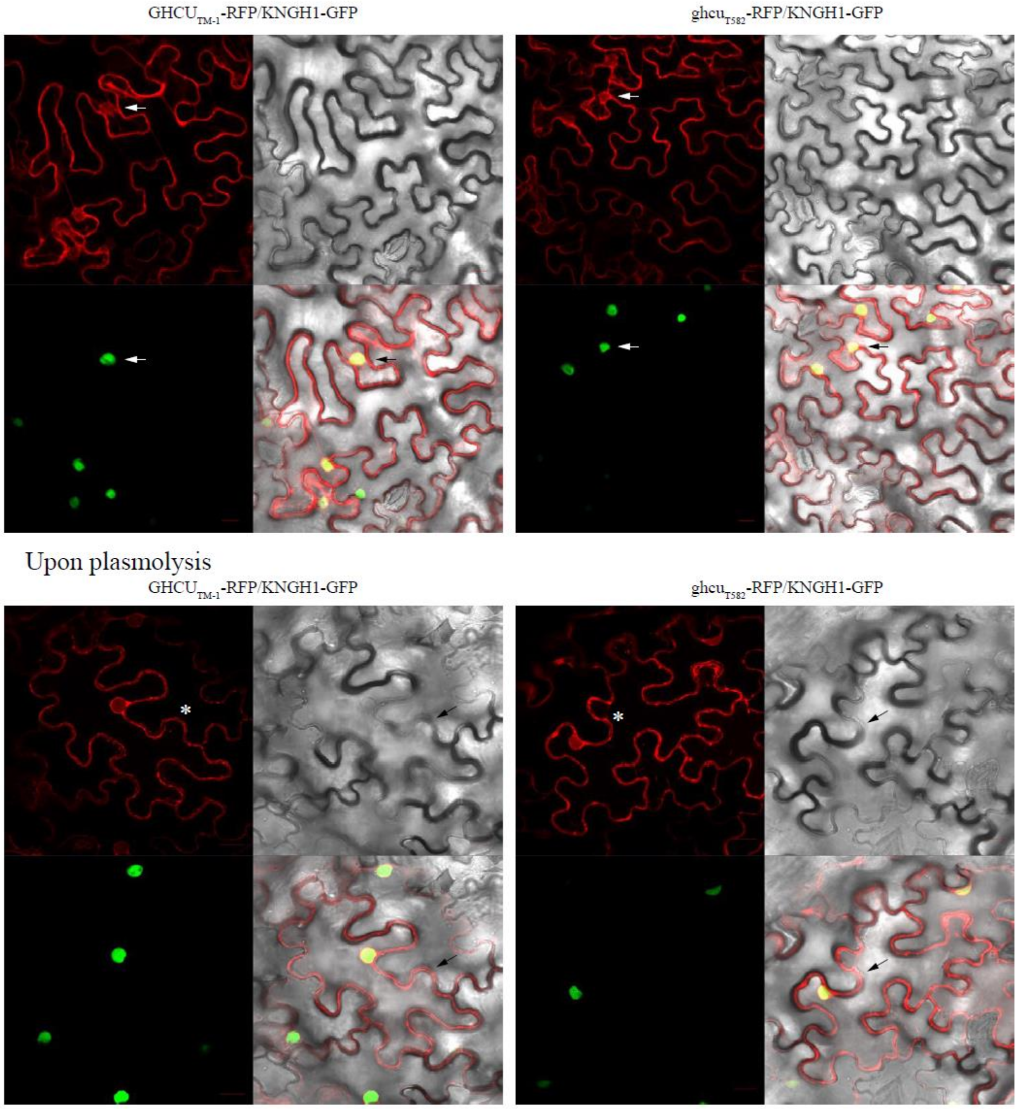

Fig. S11 GHCU is not enriched at the PD. Plasmolysis in the leaf epidermis does not show any noticeable enrichment of GHCU:GFP in the PD. The White arrow indicates the cell nucleus, while the asterisks indicate partial separation of the plasma membrane from the cell wall. Scale bars = 20 μm.

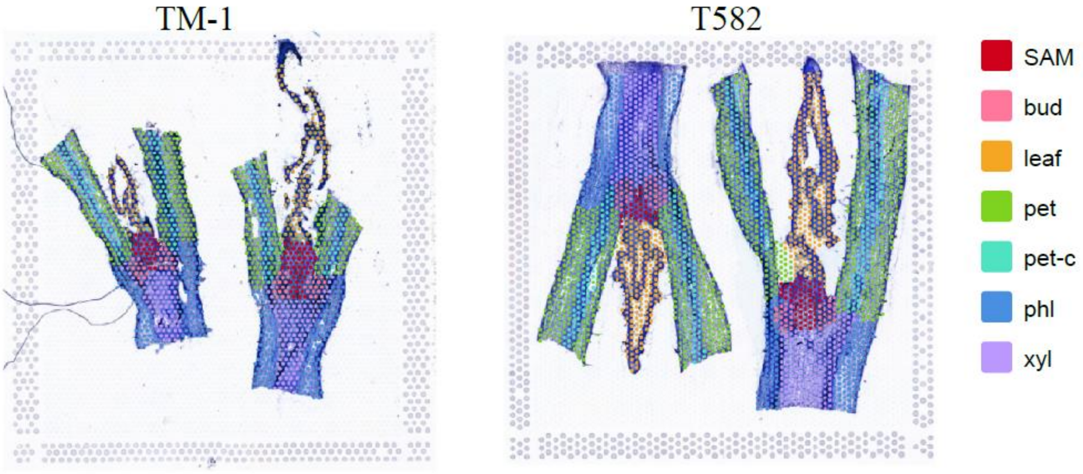

Fig. S12 Division of seven anatomical shoot tip regions.

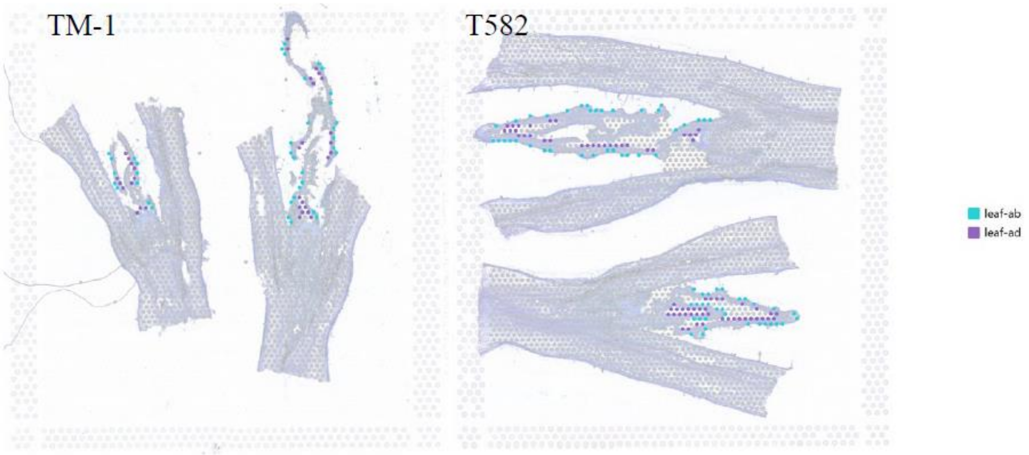

Fig. S13 The adaxial and abaxial epidermal areas are separately delineated on slices of two samples, leaf-ab: the SPOTs were chosen for abaxial side; leaf-ad: the SPOTs were chosen for adaxial side.

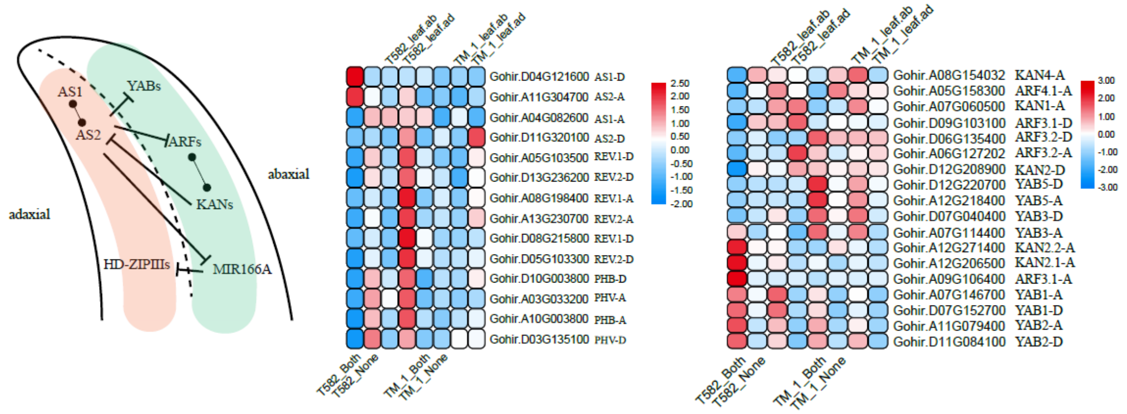

Fig. S14 Heat map of the reported abaxial-adaxial marker genes, including AS1, AS2, HD-ZIPIII, YAB1/2/3/5, ARF3/4 and KAN2/4.

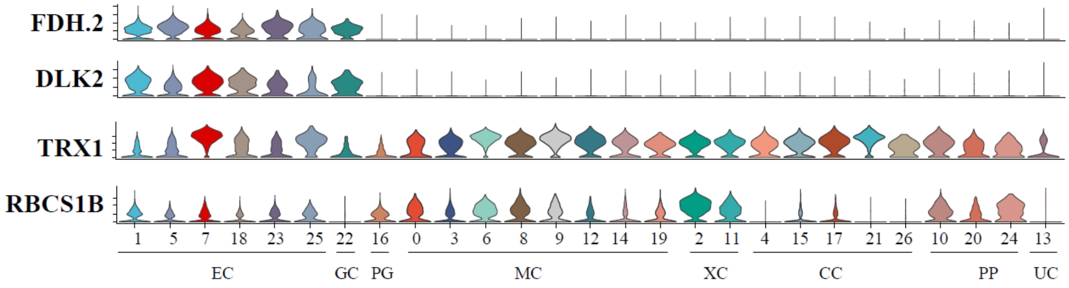

Fig. S15 Violin plots showing the expression pattern of representative cell-specific marker genes used to assign cell types.
